# Integrative transcriptomic analysis suggests new autoregulatory splicing events coupled with nonsense-mediated mRNA decay

**DOI:** 10.1093/nar/gkz193

**Published:** 2019-02-27

**Authors:** Dmitri Pervouchine, Yaroslav Popov, Andy Berry, Beatrice Borsari, Adam Frankish, Roderic Guigó

**Affiliations:** 1 Skolkovo Institute of Science and Technology, Ulitsa Nobelya 3, Moscow 121205, Russia; 2 Faculty of Bioengineering and Bioinformatics, Moscow State University, Leninskiye Gory 1-73, 119234 Moscow, Russia; 3 European Molecular Biology Laboratory, European Bioinformatics Institute, Wellcome Genome Campus, CB10 1SA Hinxton, Cambridge, UK; 4 Center for Genomic Regulation, The Barcelona Institute of Science and Technology, Dr. Aiguader 88, Barcelona 08003, Spain; 5 Universitat Pompeu Fabra (UPF), Barcelona 08003, Spain

## Abstract

Nonsense-mediated decay (NMD) is a eukaryotic mRNA surveillance system that selectively degrades transcripts with premature termination codons (PTC). Many RNA-binding proteins (RBP) regulate their expression levels by a negative feedback loop, in which RBP binds its own pre-mRNA and causes alternative splicing to introduce a PTC. We present a bioinformatic analysis integrating three data sources, eCLIP assays for a large RBP panel, shRNA inactivation of NMD pathway, and shRNA-depletion of RBPs followed by RNA-seq, to identify novel such autoregulatory feedback loops. We show that RBPs frequently bind their own pre-mRNAs, their exons respond prominently to NMD pathway disruption, and that the responding exons are enriched with nearby eCLIP peaks. We confirm previously proposed models of autoregulation in SRSF7 and U2AF1 genes and present two novel models, in which (i) SFPQ binds its mRNA and promotes switching to an alternative distal 3′-UTR that is targeted by NMD, and (ii) RPS3 binding activates a poison 5′-splice site in its pre-mRNA that leads to a frame shift and degradation by NMD. We also suggest specific splicing events that could be implicated in autoregulatory feedback loops in RBM39, HNRNPM, and U2AF2 genes. The results are available through a UCSC Genome Browser track hub.

## INTRODUCTION

Gene expression in higher eukaryotes is regulated at many different levels. The output of the transcriptional program is maintained by a large number of protein factors and *cis*-regulatory elements, which control the balance between mRNA production and degradation (1,2). Nonsense mutations and frame-shifting splicing errors induce premature termination codons (PTC) that give rise to mRNAs encoding truncated, dysfunctional proteins. In eukaryotic cells, mRNA transcripts with PTC are selectively degraded by the surveillance mechanism called Nonsense-Mediated mRNA Decay (NMD) (3).

The so-called exon junction complex-dependent (EJC) model postulates that NMD distinguishes between normal and premature translation termination in the cytoplasm, where ribosomes displace EJCs from within, but not downstream of the reading frame (4,5). These complexes are deposited ∼20–24 nucleotides (nt) upstream of the exon-exon junctions during pre-mRNA splicing (6). EJCs that remain associated with the mRNA after the initial round of translation serve as indicators of whether the termination codon is premature or not, because the normal termination codons are usually located in the last exon. The presence of EJCs 50–55 nucleotides downstream of the stop codon triggers a cascade of events, in which the up-frameshift 1 factor (UPF1) plays a central role (5). The phosphorylated UPF1 recruits the endonuclease SMG6 and other factors causing deadenylation and decapping, targeting the cleaved mRNA for degradation by cellular exonucleases (5). Other models propose that sensing the distinction between a normal termination codon and a PTC depends on the distance between the terminating ribosome and the poly(A) tail, in which the interaction of eRF3 with PABPC1 is important, or that an early ribosome release caused by the PTC exposes the downstream unprotected mRNA to degradation by nucleases independently of EJCs (7).

It has been increasingly reported over past years that NMD is not only dedicated to the destruction of PTC-containing mRNAs that appear as a result of nonsense mutations or splicing errors, but that it also plays a key role in regulating the expression of a broad class of physiological transcripts (8,9). Targets of NMD include tissue-specific transcripts (10), transcripts with mutually exclusive exons (11), mRNAs with upstream open reading frames (uORFs) and long 3′-untranslated regions (UTRs) (12), and transcripts emanating from transposons and retroviruses (13). The mechanism, in which the cell employs alternative splicing (AS) coupled with NMD to downregulate the abundance of mRNA transcripts, shortly termed AS-NMD (14) (also referred to as regulated unproductive splicing and translation (8) or unproductive splicing (15)), is found in all eukaryotes that have been studied to date and often exhibits a high degree of evolutionary conservation (16,17). Current estimates by the GENCODE consortium indicate that up to one-third of human protein-coding genes have at least one annotated transcript isoform with a splice site >50 nts downstream of the end of the coding sequence, and that a significant fraction of them have mammalian orthologs (18). These predictions suggest that unproductive splicing is a widespread and functionally selected mechanism of post-transcriptional control of gene expression.

NMD is involved in the development of cancers, where it can downregulate the expression of tumor-suppressor genes or activate the expression of oncogenes that are normally suppressed (19). In several cancers including hepatocellular carcinoma, alternative splicing of the Kruppel-like factor 6 (KLF6) tumor suppressor gene leads to a pathogenic AS-NMD splice variant associated with increased tumor metastasis and mortality (20). Inhibition of the NMD pathway stabilizes many transcripts necessary for tumorigenesis (21). Intriguingly, a substantial number of long non-coding RNAs are also found to be substrates of NMD, suggesting that NMD pathway is not exclusively dedicated to mRNAs (22–24). Among them is the metastasis-associated lung adenocarcinoma transcript 1 (MALAT1), which is downregulated in gastric cancer upon UPF1 overexpression (25).

Many RNA-binding proteins (RBPs), and particularly splicing factors (SFs), control their own expression levels by a negative feedback loop mediated by AS-NMD, in which the excessive amount of RBP binds its own pre-mRNA and causes alternative splicing to induce a PTC. Studies have shown that mutations in RBP binding sites abolish this negative feedback, while RBP overexpression leads to the increased fraction of unproductively-spliced mRNA (26). To date, many genes that utilize this mechanism are known, including SR proteins (15,27,28), hnRNP family members (29–31), TDP-43 (32,33), TRA2β (34), MBNL (35,36), PTB (37), CHTOP (38), FUS (39) and core spliceosomal and ribosomal proteins (40–42). Besides autoregulation, some RBPs use AS-NMD to cross-regulate the expression of their family members. Examples include hnRNPL/hnRNPLL (31), PTBP1/PTBP2 (37,43), RBM10/ RBM5 (40), RBFOX2/PTBP2 (44), RBFOX2/RBFOX3 (45) and others. Disruption of auto- or cross-regulation of SFs is associated with human pathogenic states, including neurodegenerative diseases and cancer (40,46–48).

**Figure 1.**
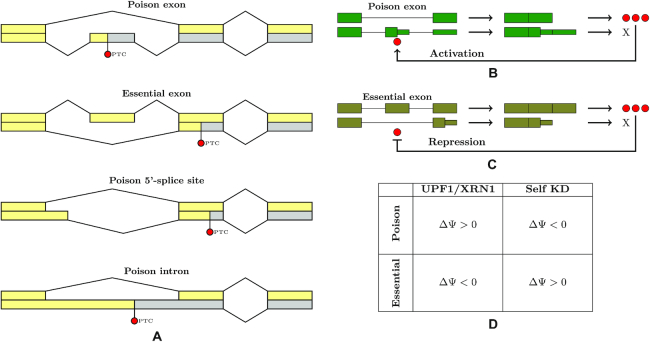
(**A**) The relationship between alternative splicing events and the induced PTC. Top-down: a poison exon causes NMD when included; an essential exon causes NMD when skipped; a poison 5′-splice site causes a frame shift and induces a downstream PTC; a retained poison intron carries a PTC. (**B**) RBP binding to its own pre-mRNA promotes inclusion of a poison exon and creates a negative feedback loop; shRNA-KD of an activating RBP should promote exon skipping. (**C**) RBP binding to its own pre-mRNA promotes skipping of an essential exon; shRNA-KD of a repressing RBP should promote exon inclusion. (**D**) A summary of the expected splicing changes in poison and essential exons after NMD inactivation and after the depletion of RBP itself.

The connection between AS event and the position of PTC that is induced by it is not always evident because PTC may appear anywhere downstream. The simplest and the most studied case is the so-called poison cassette exon, i.e. cassette exon with an early in-frame PTC, which is normally skipped, but triggers degradation by NMD when included in the mature mRNA (15) (Figure 1a). The classic examples of auto- and cross- regulatory poison exons are documented in serine/arginine-rich (SR) proteins (15). However, other classes of AS events such as alternative 5′- and 3′-splice sites or intron retention also contribute to AS-NMD (49). A case that is reciprocal to poison exons, termed here as “essential” exon, occurs when a cassette exon, which is normally included in the mature mRNA, triggers NMD when skipped. For instance, alternative skipping of PTB exon 11 yields an mRNA that is removed by NMD, and the skipping is itself promoted by PTB in a negative feedback loop (37). Similarly, the spliceosomal RNA binding protein RBM10, which is associated with TARP syndrome and lung adenocarcinoma, downregulates its own expression and that of RBM5 by promoting skipping of several its essential exons (40).

In this work, we systematically search for RBPs with autoregulatory AS-NMD feedback loops by combining three publicly available data sources: the enhanced crosslinking and immunoprecipitation (eCLIP) assay for a large panel of RBPs (50), short-hairpin RNA (shRNA) knockdowns (KD) of the same set of RBPs followed by RNA sequencing (RNA-seq) (51–53), and shRNA co-depletions of UPF1/XRN1 and SMG6/XRN1 followed by RNA-seq (24). We chose to analyze these data despite they come from different human cell lines (K562 and HepG2 for eCLIP and RBP-KD, and HEK293 for NMD inactivation) expecting that AS-NMD programs must be shared between conditions as they control the core of cellular homeostasis. The eCLIP peaks provide information about RBP binding at different transcriptomic locations, while shRNA-KD experiments measure the changes in exon inclusion levels (ΔΨ; see Materials and Methods) for all expressed exons genomewide.

The logic of our approach is that in order to autoregulate its expression, an RBP should bind its own pre-mRNA, i.e., it should contain a cognate eCLIP peak in its own gene. Second, in response to the inactivation of NMD pathway, poison exons, which are suppressed by it, should increase their inclusion levels (ΔΨ > 0), while essential exons, skipping of which is normally suppressed, should conversely decrease their inclusion level (ΔΨ < 0). Third, the autoregulation via AS-NMD can be achieved by either activating (Figure 1B) or repressing (Figure 1C) mechanisms. In the former case, the excess of RBP may activate the inclusion of a poison exon in its pre-mRNA, which would lead to the degradation of that spliced mRNA by NMD. Then, shRNA-KD of an activating RBP should lead to a lower inclusion rate of the poison exon (ΔΨ < 0). Conversely, the excess of RBP may suppress the inclusion of an essential exon, also leading to the degradation by NMD. Then, shRNA-KD of a repressive RBP should result in a higher inclusion level of the essential exon (ΔΨ > 0). That is, poison and essential exons should react oppositely to the depletion of their host genes and to that of NMD pathway. These anticorrelated changes are summarized in Figure 1D.

The rest of the paper is organized as follows. We first show statistically that RBPs with annotated NMD transcripts tend to bind their own pre-mRNAs more frequently than do other RBPs. Next, we demonstrate that poison and essential exons react oppositely to the disruption of NMD pathway and tend to co-occur with binding sites of their cognate RBPs. Using stringent thresholds, we identify a set of exons with nearby cognate eCLIP peaks that significantly and substantially change their inclusion level in response to NMD pathway inactivation and to downregulation of their host genes. This allows to propose specific regulatory mechanisms for a number of genes including serine/arginine-rich and proline/glutamine-rich splicing factors SRSF7 and SFPQ, human ribosomal protein RPS3, and spliceosomal auxiliary factor U2AF1, as well as for several lower-confidence predictions, including RBM39, HNRNPM and U2AF2. The results of integrative analysis, including predictions at less stringent cutoffs, are organized as a track hub for UCSC Genome Browser (see Materials and Methods).

## MATERIALS AND METHODS

### Genomes and transcript annotations

February 2009 assembly of the human genome (hg19, GRCh37) was downloaded from Genome Reference Consortium (54). The respective transcript annotation v19 was downloaded from GENCODE website (55). Transcript annotations were parsed to extract positions of introns and exons. Intron and exon sequences were extracted using bedtools getfasta tool (56). A transcript was considered as NMD target if it was labeled as ‘nonsense_mediated_decay' by GENCODE, i.e. if the coding sequence of the transcript finishes >50 nts from a downstream splice site. A human protein-coding gene will be referred to as Gene With NMD (GWN) if it contains at least one transcript isoform annotated as NMD (18), and Gene Without NMD (GWO) otherwise.

An annotated exon [*x, y*] with the acceptor site *x* and the donor site *y* is defined to be a cassette exon if there exist introns [*a, x*] and [*y, b*] such that [*a, b*] is also an intron, i.e. the intervals [*a, x*], [*y, b*], and [*a, b*] are introns in at least one annotated transcript. A cassette exon is defined to be a poison exon, if it contains a stop codon of an annotated NMD-transcript. For essential exons, we use a different definition to avoid connecting exon skipping to the induced downstream PTC. For each exon in each annotated CDS, we check whether it is essential or not by removing its nucleotide sequence from the transcript, translating the modified nucleotide sequence to aminoacids, and checking if a PTC appears >50 nts upstream of at least one splice junction. A relative position of an exon in a transcript is defined as the position of its midpoint normalized by a linear transformation to the range from 0% to 100%, where the 5′-exon is 0% and the 3′-exon is 100%.

### RNA-binding proteins (RBP)

The list of genes with an annotated RNA-binding function was obtained by searching for the term ‘RNA binding' (GO:0003723) in the table that was obtained by merging ENSEMBL identifiers of human genes with Gene Ontology annotation on UniProt identifiers (57,58). The resulting list of RBPs containing 1544 genes is shown in Supplementary Data File 1.

### Enhanced crosslinking and immunoprecipitation assay (eCLIP)

We used publicly available eCLIP data for 115 RBPs profiled in (50). eCLIP peaks, which were called by the data producers, were downloaded from ENCODE data repository in bed format (52,53). The peaks in two immortalized human cell lines, K562 and HepG2, were filtered by the condition log FC ≥ 3 and *P*-value <0.001 as recommended (50). Since the agreement between peaks in the two replicates was moderate (the median Jaccard distance 25% and 28% in K562 and HepG2, respectively), we took the union of peaks between the two replicates within each cell line, and then pooled the resulting peaks between cell lines. A summary of eCLIP profiles that were used in this study and their accession numbers is given in Supplementary Table S1.

### Short-hairpin RNA knockdown of RBP followed by RNA-seq

Publicly available data on short-hairpin (shRNA) knockdown of human RBPs followed by RNA-seq (shRNA-KD) (51) were downloaded in BAM format from ENCODE data repository (52,53). A summary of RBP depletion data and the respective accession numbers is given in Supplementary Table S1. Exon inclusion metrics (PSI) were called for all annotated exons using IPSA software with the default settings (59). PSI values of individual exons (see below) were computed based on split-read counts that were pooled between bioreplicates.

### NMD pathway inactivation

We used the expression profiling by RNA-sequencing in HEK293 Flp-In T-Rex cells that were subjected to siRNA-mediated depletion of XRN1 and co-depletion of either UPF1 or SMG6 (GEO accession GSE57433) (24). Transcript quantification for target datasets (GSM1382448 for UPF1 and GSM1382447 for SMG6) versus control (GSM1382445) were done using cufflinks2 by data producers (60). The resulting read counts were processed in R statistics software by DESeq2 package using normal shrinkage correction (61). Due to the fact that the original data was not replicated, the corrected *P*-values for gene expression were not computed. To quantify splicing changes, we remapped the original data to the human genome using STAR aligner version 2.5.3a and applied IPSA software with the default settings to compute the number of split reads supporting exon inclusion and exclusion (59).

### Exon inclusion rate (Ψ)

Genomic alignments of short reads from all RNA-seq experiments were processed using IPSA pipeline to obtain read counts supporting splice junctions (59). Read counts were filtered by the entropy content of the offset distribution, annotation status and canonical GT/AG dinucleotides at splice sites (59). The exon inclusion rate (Ψ, Percent-Spliced-In, or PSI ratio) was calculated for exons of annotated protein-coding and NMD transcripts according to the equation

\begin{equation*} \Psi = \frac{inc}{inc+2*exc},\nonumber \end{equation*}

where *inc* is the corrected number of reads supporting exon inclusion and *exc* is the corrected number of reads supporting exon exclusion. Ψ values with the denominator below 20 counts were considered unreliable and discarded. This definition of exon inclusion rate was applied not only to cassette exons, but also to other types of AS events, e.g. alternative 5′- and 3′-splice sites, whenever inclusion and exclusion reads allow successful discrimination between alternatively-spliced transcript isoforms.

### Exon inclusion change (ΔΨ)

The change in exon inclusion rate was assessed by using ΔΨ = Ψ(*KD*) − Ψ(*Control*) metric, where Ψ(*KD*) and Ψ(*Control*) are exon inclusion rates in the KD experiment and in the control, respectively. In order to account for the inherent problem of measuring alternative splicing of transcripts that are themselves reduced in levels by shRNA-KD, we used the number of reads in the denominator of Ψ as a proxy for the local gene expression level at a given exon and subtracted the confounding effect of gene expression using a regression model. Namely, we computed log_10_(*FC*), where *FC* is the fold change in the combined number of split reads supporting exon inclusion and exclusion between KD and control and built a linear model of the form ΔΨ_*i*_ = β_0_ + β_1_log_10_(*FC*_*i*_) + *e*_*i*_ for all exons with ΔΨ ≠ 0. The values of β_1_ were positive numbers in the range from 0.001 to 0.025 (per order of magnitude of *FC*), thus confirming that exon inclusion rates tend to increase with increasing gene expression level. We therefore removed the confounding effect of gene expression on exon inclusion by replacing each ΔΨ value by its residual in the linear model.

Using the same logic, we developed a procedure to assess statistical significance of exon inclusion changes correcting for the local gene expression level using as a proxy the number of reads in the denominator of Ψ. The distribution of ΔΨ values spans the interval from −1 to 1 and, as in the case of gene expression, its mean and standard deviation depend on gene expression level. We binned Ψ values of all exons by log_10_ of the mean split read count, which is the average of Ψ denominators between KD and the control. In each bin, we computed the mean and the standard deviation of ΔΨ (exons with ΔΨ = 0 were excluded) and assigned the corresponding *z*-score to each exon. The distribution of *z*-scores was bell-shaped (Supplementary Figure S1) and it was not unreasonable to assign a normal one-tail probability to each exon. In order to estimate the false discovery rate in multiple tests, the *P*-values were transformed into *q*-values correcting for the total number of exons in the respective test.

### Gene ontology analysis

We used Human Gene Ontology annotation provided by Gene Ontology (GO) Consortium (57,62). Enrichment of GO terms in gene sets of interest was done using GOstats library (63) within Bioconductor R package, and also using GOrilla, a tool for GO term enrichment analysis in ranked gene lists (64).

### Statistical analysis

The data were analyzed and visualized using R statistics software version 3.4.1 and ggplot2 package. The test for proportions, referred to as π-test, was performed using normal approximation to binomial distribution for samples of size *n* > 40 without continuity correction. Non-parametric tests were performed by built-in R functions using normal approximation with continuity correction. Wilcoxon one-sample test was used to assess ΔΨ distribution for departures from zero. One-sided *P*-values are reported throughout the paper.

In order to control the false discovery rate (FDR) in multiple hypothesis testing, the *P*-values were transformed into *q*-values using qvalue R package version 2.15.0 (65). The −log_10_(*q*)-values in the integrated prediction set were estimated as the sum of the respective −log_10_(*q*) values for NMD depletion, shRNA-KD of the host RBP, and eCLIP (which is equivalent to taking the product of *q*-values). In Supplementary Material, *P*- and *q*-values are presented in −log_10_ scale.

### Software

The pipeline described in this article is implemented using GNU make utility. The code is available at the github repository (https://github.com/pervouchine/nmdnar). The pipeline generates a track hub file for the UCSC Genome Browser (66), which is available through the following URL https://raw.githubusercontent.com/pervouchine/nmdnar/master/hub/hub.txt.

## RESULTS

### RBP often undergo NMD and bind their own pre-mRNAs

Throughout this paper, a human protein-coding gene with at least one annotated NMD transcript is referred to as GWN (Gene With NMD); otherwise it is referred to as GWO. First, we assessed the functional attribution of GWN by Gene Ontology analysis (64). Genes with annotated molecular function of RNA-binding, nucleotide and ribonucleotide binding, and genes involved in biological processes related to splicing were significantly enriched among GWN compared to GWO (Supplementary Table S2). In what follows, we focused on the analysis of genes that are both GWN and RBP.

Approximately one third of the annotated human protein-coding genes are GWN (32%, or 6476 out of 20 242) (18). We asked whether the proportion of GWN among specific gene classes is greater than that among all protein-coding genes. Indeed, 595 out of 1544 RBPs are GWN, which is significantly greater than the background proportion (38% versus 32%, one-sample π-test, *P* = 6 × 10^−4^). This enrichment is not due to longer gene loci or to a larger number of exons in RBPs since the difference remains significant when RBPs are compared to a random sample of genes matched by the length (38% vs. 33%, two-sample π-test, *P* = 0.006) or by the number of exons (38% versus 34%, *P* = 0.01). Furthermore, 55 out of 115 splicing-related RBPs that were profiled by eCLIP are GWN, indicating a greater GWN enrichment (48% versus 32%, one-sample π-test, *P* = 3 × 10^−4^). Thus, we confirm that RBP, and particularly splicing-related RBPs, as a class of genes have a higher propensity to undergo NMD than do other protein-coding genes.

We next asked whether RBPs tend to bind their own pre-mRNAs. To test this, we analyzed eCLIP profiles of 115 RBPs and intersected them with the genomic ranges that encode their cognate genes. While 35 out of 55 GWNs profiled by eCLIP contain at least one eCLIP peak in their own gene, the respective proportion for GWO is 24 out of 60, i.e. for GWN the proportion is significantly greater (64% versus 40%, two-sample π-test, *P* = 0.005). This enrichment is not due to an imbalance in eCLIP signal density since the number of eCLIP peaks that fall within RBP genes is not significantly different between GWN and GWO (Wilcoxon test, *P* = 0.15). This indicates that RBPs with annotated NMD events tend to bind their own pre-mRNA more frequently than do RBPs without annotated NMD events. According to this statistical evidence, one may expect that RBPs frequently autoregulate their expression via AS-NMD. It is the aim of this paper to identify such cases.

### RBPs are enriched among NMD inactivation targets

The degradation of transcripts by NMD is initiated by UPF1-activated endonucleolytic cleavage of the nonsense RNA in the vicinity of the PTC followed by a rapid digestion by cytoplasmic 5′-3′ exonucleases (67,68). Specifically, the exonuclease XRN1 degrades the 3′-fragment derived from the endonucleolytic cleavage, as well as the decapped full-length nonsense RNA (67). We analyzed publicly available data on transcriptome-wide identification of NMD substrates by UPF1/XRN1 and SMG6/XRN1 co-depletions (24) and performed differential gene expression analysis. To quantify splicing changes, we computed ΔΨ metric for all exons of annotated transcripts as explained in Materials and Methods .

**Figure 2.**
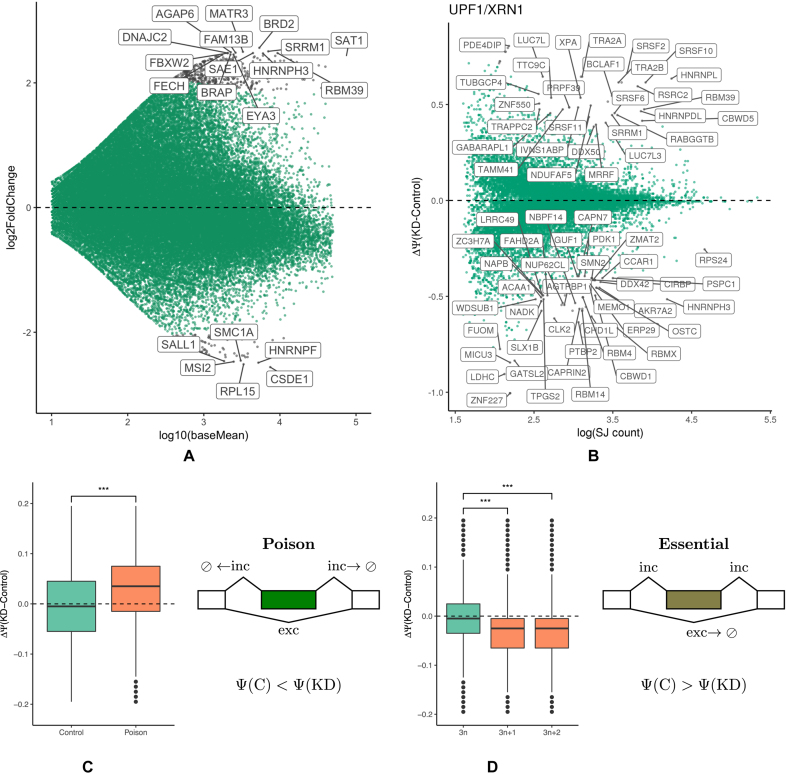
(**A**) DEseq2 analysis of differential gene expression in UPF1/XRN1 co-depletion. The top 15 overexpressed genes are shown in Supplementary Table S3. (**B**) Differential exon inclusion analysis in UPF1/XRN1 co-depletion. Changes at 0.1% FDR are shown in orange. The list of differential exons is shown in Supplementary Data File 2. (**C**) Poison exons increase their inclusion rate upon UPF1/XRN1 co-depletion. (**D**) Essential exons (3*n* + 1 and 3*n* + 2) decrease their inclusion rate upon UPF1/XRN1 co-depletion, while non-essential (3*n*) exons remain on average unchanged. Significant differences in proportions are shown by asterisks (*P < 0.01; **P < 0.05, ***P < 0.01).

The ontology analysis of genes that are substantially upregulated in UPF1/XRN1 co-depletion reveals a significant enrichment of genes related to splicing, including components of the heterogeneous nuclear ribonucleoprotein complexes (hnRNPs) and RNA-binding factors known to co-localize with core spliceosomal proteins (Figure 2A). The top 15 genes with the largest fold change and their respective GO terms are listed in Supplementary Tables S3 and S4, respectively. Although the lack of replication in the co-depletion experiment does not permit rigorous assignment of statistical significance, it can be noted that more genes are upregulated upon UPF1/XRN1 co-depletion compared to genes that are downregulated at the same value of log *FC*, indicating that the upregulated gene set contains natural NMD targets (Figure 2A).

In contrast, the analysis at the exon level (Figure 2B) reveals that a balanced proportion of exons significantly increase and decrease their level of inclusion when UPF1 and XRN1 are depleted (such exons are referred to as reactive exons). Among genes containing exons that respond to UPF1/XRN1 co-depletion again there are core spliceosomal proteins, serine-arginine rich proteins, hnRNPs, and general RNA-binding proteins (see Supplementary Table S5 for Gene Ontology analysis, and Supplementary Data File 2 for the list of ΔΨ values of reactive exons). A similar pattern is observed for the co-depletion of XRN1 with SMG6 (Supplementary Figure S2A). ΔΨ values are highly consistent between the two co-depletions (Supplementary Figure S2B; Pearson correlation *r* = 0.85). In what follows, we therefore treated UPF1/XRN1 and SMG6/XRN1 as replicates and used the average ΔΨ value, which in what follows is referred to as ΔΨ(*NMD*).

Exons that substantially change their level of inclusion are not uniformly distributed along transcripts and tend to concentrate in the 5′-end of the gene (Supplementary Figure S3). We checked whether the genes with annotated human uORFs tend to contain such exons in their 5′-ends. Indeed, 39 out 8243 exons that respond to the inactivation of NMD pathway components with |ΔΨ| ≥ 0.1 belong to the genes with annotated human uORFs from uORFdb (69), and 11 out of 863 such exons belong to the first 5% of the gene sequence, while on average 4.08 exons are expected to do so by chance (*P* = 0.001). This result is consistent with the observation in yeast that NMD frequently affects splicing events in uORFs and 3′-UTRs (12).

### Poison and essential exons react oppositely to NMD inactivation

In a broad sense, a poison (essential) exon is defined as an exon that triggers NMD when included in the mRNA (skipped, respectively). We asked whether poison and essential exons react differently to UPF1/XRN1 co-depletion. When NMD pathway is not perturbed, transcripts that contain poison exons are NMD targets. Hence, Ψ value of a poison exon should increase upon UPF1/XRN1 co-depletion because splice junctions (SJ) that support exon inclusion are degraded to a lesser extent when NMD is suppressed (Figure 2C). As expected, the distribution of ΔΨ for poison exons is significantly biased towards positive values as compared to the distribution of ΔΨ for the control set of non-poisonous cassette exons. For example, the inclusion of a poison exon that is located in the 3′-UTR of SRSF3 gene is significantly upregulated in UPF1/XRN1 knockdown with ΔΨ = 0.34 (*q* < 0.002). Note that not all annotated poison exons react positively to UPF1/XRN1 co-depletion, likely reflecting complex higher-order responses in the gene regulatory network upon NMD pathway perturbation.

Conversely, SJs supporting the exclusion of an essential exon correspond to the transcripts that are degraded by NMD. Thus, the Ψ value of an essential exon should decrease upon UPF1/XRN1 co-depletion since exon skipping products are degraded to a lesser extent when NMD is suppressed (Figure 2D). The analysis of annotated exons shows that exons of length 3*n*, where *n* is an integer, generally do not introduce PTC, while exons of length 3*n* + 1 and 3*n* + 2 lead to a frameshift that almost certainly induces a PTC (Supplementary Figure S4). That is, the majority of 3*n* + 1 and 3*n* + 2 exons are essential, while the majority of 3*n* exons are non-essential. In accordance with this, the distribution of ΔΨ for 3*n* exons is symmetric around zero, while that of ΔΨ for 3*n* + 1 and 3*n* + 2 exons significantly deviates from zero towards negative values (Figure 2d). For example, skipping of exon 10 in PTBP2 is substantially and significantly downregulated upon UPF1/XRN1 co-depletion with ΔΨ = −0.64 (*q* < 10^−9^). Of note, the corresponding transcript isoform ENST00000541987 is not yet annotated as NMD.

### Reactive exons are associated with eCLIP peaks

We next asked whether exons that react to the inactivation of the NMD pathway tend to overlap eCLIP peaks. Out of 29 336 exons that were tested, 531 exons significantly changed their inclusion level (*q* < 0.05), and 154 exons contained a cognate eCLIP peak within 200 nts. However, we observed 8 out of 531 reactive exons that contained a cognate eCLIP peak within 200 nts, while only 2.7 were expected to do so by chance (*P* = 0.002). Most of these exons (listed in Table 1) have been experimentally shown to be linked to AS-NMD, e.g. exon 6a of HNRNPL (31), exon 10 of BCLAF1 and exon 2 of TRA2A (41), exon 6 of RBM5 (40). SRSF1 and SRSF7 were predicted to contain autoregulatory poison exons (15). Note, however, that exon 4 of SRSF1 has ΔΨ < 0 that is characteristic for essential exons, which could be due existence of other exons that share a splice site with exon 4 of SRSF1, but have opposite effects on the downstream PTC. Additionally, we compared the distance to the closest cognate eCLIP peak for each exon and found that exons that significantly changed their inclusion level (*q* < 0.05) were on average 600 nt closer to cognate eCLIP peaks than were other exons (Mann–Whitney test, *P* < 10^−7^, Supplementary Figure S5), further supporting the observed positive association.

**Table 1.** Exons reactive to NMD knockdown (*q* < 0.05) with cognate eCLIP peaks within 200 nts. Some exons have two slightly different variants. Exon coordinates are in GRCh37 genome assembly.

Gene	Exon	ΔΨ_*UPF*1_	ΔΨ_*SMG*6_	−log (*q*)
SRSF1	chr17:56082759-56082961	−0.30	−0.15	1.6
HNRNPL	chr19:39332253-39332322	0.64	0.48	16.0
SRSF7	chr2:38976040-38976488	0.28	0.20	1.9
RBM5	chr3:50137965-50138038	−0.29	−0.29	1.6
BCLAF1	chr6:136590279-136590348	0.52	0.46	13.9
BCLAF1	chr6:136590279-136590441	0.55	0.47	15.5
TRA2A	chr7:23561751-23562051	0.65	0.49	15.4
TRA2A	chr7:23561973-23562051	0.65	0.51	15.6

Relaxing the threshold on FDR while requiring large effect size expectedly leads to a broader set of predictions, among which we find other known AS-NMD events, e.g. an essential exon in FUS gene (39) with ΔΨ = −0.16 (*q* ≃ 0.8) as well as several unknown, but plausible candidates, e.g. an essential exon in HNRNPM gene with ΔΨ = −0.13 (*q* ≃ 0.7) and a poison exon in U2AF2 gene with ΔΨ = −0.15 (*q* ≃ 0.6) (Supplementary Figures S6A-C). However, it is unclear what proportion of these predictions are false positives. In order to obtain a list of high-confidence candidates, we combined the list of reactive exons with a nearby cognate eCLIP peak and a similar list obtained in differential splicing analysis of shRNA-KD for a large panel of RPBs (51), which is discussed in the next section.

### Regulatory exons react to the depletion of their host genes

Perturbations of RBP expression must affect autoregulatory mechanisms that sense cellular RBP concentrations. Since RBP binding may exert both activating and repressing effects on exon inclusion, the autoregulation via AS-NMD can be achieved by either activating (Figure 1B) or repressing (Figure 1C) mechanisms. In the former case, shRNA-KD of an activating RBP should lead to a lower inclusion rate of the poison exon, i.e. ΔΨ of such exon should be negative. In the latter case, shRNA-KD of a repressive RBP should result in a higher inclusion level of the essential exon, i.e., ΔΨ of such exon should be positive. The patterns of expected ΔΨ values are opposite for poison and essential exons, and also opposite for each exon in perturbations of its host gene and in NMD pathway perturbations (Figure 1D).

To apply this reasoning to the identification of novel AS-NMD autoregulatory targets, we computed ΔΨ between KD and control experiments (51) for 150 RBPs (Supplementary Table S1), again correcting for the changes in the host gene expression level. First, we tested a few genes with known unproductive splicing. Earlier studies have demonstrated that alternative skipping of exon 11 in Polypyrimidine Tract Binding Protein 1 (PTBP1) leads to an mRNA that is removed by NMD, and that this mechanism degrades a large part of the PTBP1 transcripts in HeLa cells (37). While exon 11 is essential, the changes in its inclusion rate that are observed upon UPF1/XRN1 and PTBP1 depletion are small by the absolute value (ΔΨ_UPF1_ = −0.04 and ΔΨ_PTBP1_ = 0.03, respectively) and insignificant (*q* ≃ 1), but their opposite signs are consistent with the principle illustrated in Figure 1C. Interestingly, there is an eCLIP peak near exon 11 of PTBP1, but the two variants of exon 9 are reactive to PTBP1 depletion with much higher ΔΨ (Supplementary Figure S7A) suggesting that skipping of exon 11 may be masked by nearby alternative splicing events.

Another example is human TAR DNA-binding protein (TARDBP, TDP-43), which controls its own expression level through a negative feedback loop, in which it binds to the 3′-UTR in its own mRNA and induces AS-NMD (32,33). Indeed, its 3′-UTR contains a number of unproductive splicing events that become unrepressed upon NMD inactivation (Supplementary Figure S7B). The 3′-UTR of TARDBP contains several cognate eCLIP peaks, which confirm that TARDBP is capable of binding its own 3′-UTR, and a poison exon with ΔΨ = 0.25 (*q* < 0.05). However, there are no significant splicing changes in the 3′-UTR in response to TARDBP shRNA-KD (the expected change is ΔΨ < 0), which could be related to the efficacy of shRNA-KD.

**Figure 3.**
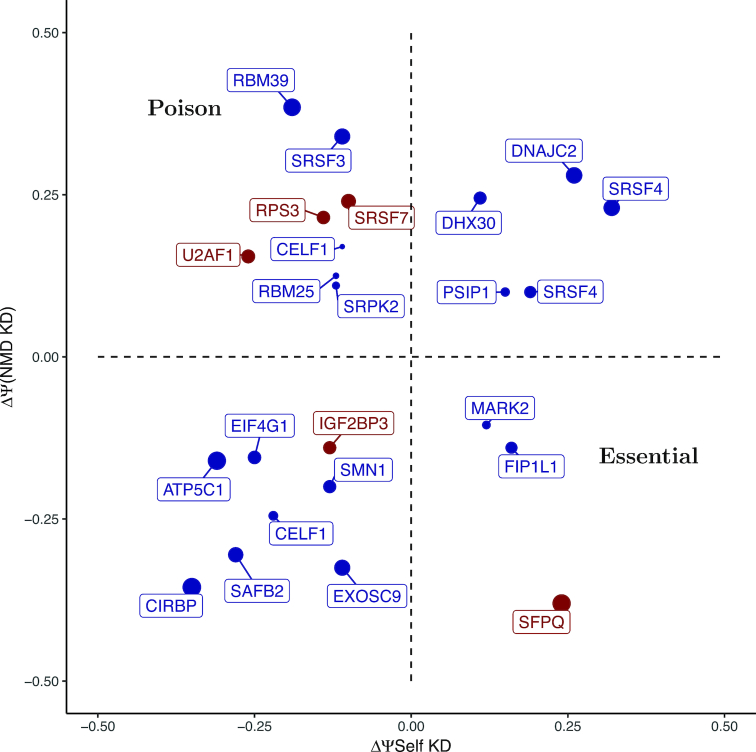
Bivariate analysis of significant splicing changes under UPF1/XRN1 co-depletion (y axis) vs. shRNA-KD of RBP itself (x-axis). Only genes with |ΔΨ| ≥ 0.1 in both axes are plotted. Genes with at least one cognate eCLIP peak are shown in red. The size of the dot is proportional to the log_10_ of the combined *q*-value, which is equal to the sum of −log_10_(*q*)-values for NMD depletion, shRNA-KD of the host RBP, and the respective eCLIP.

Since several known AS-NMD targets appear as false negatives in this analysis, we have no other choice than to go above the standard FDR cutoff of 5% and rank the predictions based on their *P*-values and magnitude of the effect size. To do so, we integrated the three data sources and applied the established cutoff value of |ΔΨ| ≥ 0.1 for both NMD inactivation and shRNA-KD of the RBP (70,71) (master table with all the predictions is available in Supplementary Data File 3). Additionally, we imposed a requirement that the mRNA of the RBP must contain at least one cognate eCLIP peak located within 5 kb from the reactive exon. As a result, the most stringent prediction set (Figure 3) contained three candidate poison exons (SRSF7, U2AF1 and RPS3), one candidate essential exon (SFPQ), and one exon (IGF2BP3) which is neither poison, nor essential because it exhibits splicing changes of the same sign. In the next section, we discuss potential AS-NMD autoregulatory mechanisms for these genes in detail.

### Case studies

**Figure 4.**
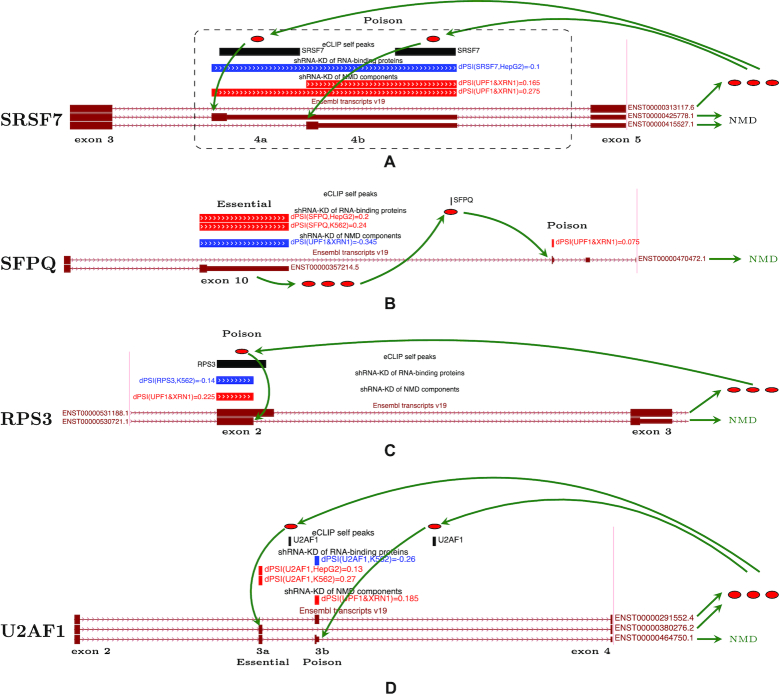
Case studies of poison and essential exons. Changes of exon inclusion levels are colored so that ΔΨ > 0 are red and ΔΨ < 0 are blue; eCLIP peaks are shown in black. (**A**) SRSF7 binds its pre-mRNA to promote the inclusion of its poison exons 4a and 4b. (**B**) SFPQ binds its pre-mRNA co-transcriptionally and switches splicing towards the alternative 3′-UTR, which is an NMD substrate. (**C**) RPS3 binds its pre-mRNA around its exon 2 and suppresses splicing at the endogenous donor site by activating an upstream donor site, which leads to a frame shift and degradation by NMD. (**D**) U2AF1 contains two mutually exclusive variants of exon 3. If exon 3a is included, the inclusion of exon 3b leads to a frame shift and NMD. According to eCLIP, U2AF1 binds its pre-mRNA around exons 3a and 3b, which leads to their simultaneous inclusion, frame shift, and NMD.

To visualize the predictions, we created a track hub for the UCSC Genome Browser (66), in which exons with ΔΨ > 0 are denoted by red color, exons with ΔΨ < 0 are denoted by blue color, and black boxes represent eCLIP peaks. In what follows (e.g. diagrams in Figure 4) we add green arrows to Genome Browser diagrams to indicate the proposed mechanism of autoregulation by AS-NMD.

#### SRSF7

Also known as splicing factor 9G8, is a member of serine/arginine-rich splicing factor family, which is important for regulation of pre-mRNA splicing, nuclear export, and translation (72). It has been reported recently that SRSF7 plays a major role in proliferation of cancer cells and apoptosis (73,74). Other serine/arginine-rich splicing factors, for example SRSF3, were shown to modulate their own alternative splicing, as well as that of other transcripts encoding SR proteins (75). Previous comparative analysis predicted that SRSF7 is implicated in a negative autoregulatory feedback loop (15). Here, we confirm the existence of two poison exons in this gene (Figure 4A). Indeed, the inclusion of exons 4a and 4b becomes substantially elevated upon UPF1/XRN1 co-depletion (ΔΨ = 0.24 with *P* < 10^−6^ and ΔΨ = 0.14 with *P* < 0.01, respectively), confirming that they both are poison exons. On the other hand, the depletion of SRSF7 by shRNA-KD promotes skipping of these exons (ΔΨ = −0.10 with *P* < 0.02 for exon 4a and ΔΨ = −0.05 with *P* < 0.06 for exon 4b), which indicates that their inclusion was activated by SRSF7 itself. Additionally, there are two eCLIP peaks located in the two poison exons. Therefore, we have a strong evidence for AS-NMD, one in which the excess of SRSF7 protein binds its own pre-mRNA to promote exon 4a and exon 4b inclusion, thus regulating its level of expression via AS-NMD.

#### SFPQ

A member of another family of splicing factors, proline/glutamine-rich splicing factor SFPQ, is associated with amyotrophic lateral sclerosis (76), Alzheimer’s Disease and Frontotemporal Dementia (77). We propose the following mechanism of SFPQ autoregulation by AS-NMD (Figure 4B). Exon 10, the terminal exon of the major SFPQ transcript isoform, is substantially downregulated in UPF1/XRN1 co-depletion (ΔΨ = −0.38 with *P* < 10^−13^), and it is also greatly upregulated when SFPQ itself is depleted (ΔΨ = 0.241 with *P* < 0.005), following the anticorrelated splicing pattern for essential exons (Figure 1c). We also noted that when exon 10 is suppressed, SFPQ splicing switches to a group of exons in the 3′-UTR, which are substantially upregulated in UPF1/XRN1 co-depletion, thus likely being poison exons. The intron spanning between exon 9 and the downstream poison exons contains a cognate eCLIP peak of SFPQ, indicating that alternative splicing and polyadenylation may be regulated by SFPQ itself. Indeed, examples of coupling between splicing and polyadenylation are not rare (78). We therefore hypothesize that SFPQ binds its own pre-mRNA downstream of exon 10 and promotes alternative splicing to the distal 3′-UTR, which contains a PTC upstream of a splice junction and thus is a substrate of NMD.

#### RPS3

Human ribosomal protein S3 (RPS3) is a component of the 40S ribosomal subunit that is mainly associated with protein synthesis. However, RPS3 has many additional extraribosomal functions and is involved in apoptosis and tumorigenesis (79). Studies in *Caenorhabditis elegans* have shown that it is not uncommon among ribosomal proteins to be AS-NMD targets (26). In particular, ribosomal proteins L3, L10a and L12 use an evolutionarily-conserved pathway to insert PTCs in their pre-mRNA (79–81). Here, we show that exon 2 of RPS3 has an alternative upstream donor site that induces a frameshift and targets RPS3 pre-mRNA for degradation (Figure 4C). This shorter variant of exon 2 reacts positively to UPF1/XRN1 co-depletion (ΔΨ = 0.22 with *P* < 0.005), confirming that the transcript with a frameshift is indeed a NMD target, and reacts negatively to RPS3 depletion (ΔΨ = −0.14 with *P* < 0.1), indicating that RPS3 is involved in promoting its inclusion. Consistently with this, exon 2 of RPS3 pre-mRNA contains a cognate eCLIP peak of RPS3, suggesting autoregulatory negative feedback loop with a poison 5′-splice site in this gene. Remarkably, the ribosomal protein L10a in *Caenorhabditis elegans* uses exactly the same strategy of regulation by specifically switching the donor site of its intron 3 to create an unproductively spliced mRNA (81).

#### U2AF1

U2 small nuclear RNA auxiliary factor 1 (U2AF1, also known as U2AF35) is another member of the serine/arginine-rich splicing factor family (82). It encodes the small subunit of U2 auxiliary factor, a basic component of the major spliceosome which mediates binding of U2 snRNP to the pre-mRNA branch site (82–84). U2AF1 is frequently mutated in cancers, particularly in myelodysplastic syndromes, along with other mutated splicing factors (85). In humans, exon 3 of U2AF1 exists in two mutually exclusive variants, exon 3a and exon 3b (Figure 4D). These exons are homologous (68.2% sequence identity) and have the same length of 67 nt, which suggests that they have evolved through a tandem genomic duplication (86). Since 67 is not a multiple of three, simultaneous inclusion of exons 3a and 3b, or simultaneous skipping of both, leads to a frameshift. Studies have reported that simultaneous inclusion of these two exons indeed leads to the degradation of the corresponding transcript isoform by NMD (11). We find that exon 3b, which is located downstream of exon 3a, reacts positively to UPF1/XRN1 co-depletion (ΔΨ = 0.18 with *P* < 10^−3^), and negatively to U2AF1 depletion (ΔΨ = −0.244 with *P* < 0.05), suggesting that it is, in fact, a poison exon. Consistently with this, two eCLIP peaks are present downstream of exons 3a and 3b suggesting that U2AF1 binding may promote inclusion of both these exons, thereby creating a negative feedback loop of AS-NMD.

#### Other genes

Besides exons that contain a nearby eCLIP peak and expectedly react to the inactivation of NMD pathway and to the depletion of their host genes, Figure 3 also contains other predictions. For example, exon 3 of RNA Binding Motif Protein 39 (RBM39) reacts positively to NMD depletion and negatively to the depletion of RBM39, thus likely being a poison exon (Supplementary Figure S8A). Indeed, inclusion of exon 3, which is 98.6% identical at the base pair level between human and Xenopus, induces a PTC downstream (87). Therefore, it appears that exon 3 of RBM39 could indeed mediate autoregulatory feedback AS-NMD loop despite eCLIP analysis failed to identify RBM39 binding to its cognate mRNA. Another example of a false negative is exon 4 of SRSF3 which was shown to regulate its own expression through an inclusion of a PTC (88) and appeared positive in iCLIP assays (75), but is missing in eCLIP (Supplementary Figure S8B).

Among candidates in Figure 3 there are also exons with ΔΨ of the same sign in both perturbations. For example, exon 3 of Insulin Like Growth Factor 2 mRNA Binding Protein 3 (IGF2BP3) gene contains a cognate eCLIP peak within 5 kb (ΔΨ(*NMD*) = −0.16 with *P* < 0.1 and ΔΨ(*IGF*2*BP*3) = −0.13 with *P* < 0.1). Exon 3 skipping produces the isoform that is also missing the successive exon and contains a PTC in exon 5 (Supplementary Figure S6D). It is possible that IGF2BP3 binds its own pre-mRNA and induces skipping of exons 3 to promote AS-NMD. However, the negative sign of ΔΨ(*IGF*2*BP*3) indicates that gene product activates exon 3 inclusion, contrary to what is expected for essential exons. Similarly, SRSF4 contains a group of poison exons mediating AS-NMD (88), but they also react positively to SRSF4 depletion, contrary to what is expected from poison exons (Figure 3). In the next section, we discuss possible origins of these codirectional Ψ changes, which correspond to a positive feedback loop (suppression of a poison exon or activation of an essential exon), rather than to the opposite changes that are characteristic for negative feedback loops.

## DISCUSSION

Gene expression includes a wide range of regulatory mechanisms that are used by the cells to adjust the production of a specific gene in response to various inputs (1). All steps of gene expression, from the transcription initiation to the post-translational protein modification, are modulated within a sophisticated gene regulatory network, in which one regulator controls, and is itself controlled, by the expression of multiple other genes. A frequently observed pattern in such networks is the so-called self-loop, i.e. an autoregulatory feedback of a gene onto itself. Autoregulation provides a simple and, perhaps, the most robust regulatory feedback that doesn’t require any intermediate steps, allowing to sense directly the cellular concentration of a given factor. More than a half of the transcription factors in bacteria regulate their own genes by a self-loop, in which the factor binds to its own promoter and either activates or represses the transcription (89,90).

While some eukaryotic genes use autoregulatory self-loops at the transcriptional level, the expression can also be modulated post-transcriptionally (91). As an example, the binding of YBX1 to a regulatory element in the 3′-UTR of YBX1 mRNA selectively inhibits its own translation (92). However, the major way to modulate gene expression post-transcriptionally is through affecting mRNA stability (93). Particularly, AS-NMD is a mechanism of regulation by mRNA degradation, which generates transcript isoforms with PTCs and promotes mRNA elimination by NMD (93). In order for it to work through a self-loop, the gene product should be able to bind its own pre-mRNA. It is therefore not completely unexpected that RPBs are enriched among GWN, as well as among genes that autoregulate their expression via unproductive splicing.

In this work, we presented a bioinformatic analysis of a large panel of high-throughput sequencing data with the goal to identify novel cases of AS-NMD self-loops. The analysis of splicing changes (ΔΨ) upon inactivation of NMD pathway components confirmed many experimentally studied cases, thus demonstrating the proof of principle for our method. We further constrained the analysis by selecting exons that are associated with eCLIP peaks and react in a characteristic way to perturbations of their host gene expression levels (Figure 1C). However, exons in genes with known autoregulatory feedback loops, such as PTBP1 and TARDBP, showed very small and insignificant splicing changes. The major factor contributing to this discrepancy must be the efficacy of shRNA-KD, which varies greatly between RBPs. Nevertheless, we expect that our approach suffers more from the false negative rate than from the false positive rate because in the worst case of inefficient shRNA-KD we underestimate the magnitude of ΔΨ. Regarding false positive predictions, a possibility remains that the observed ΔΨ, even as large by absolute value, could result from indirect responses in gene regulatory networks, but these confounding effects should be minimized when combining several independent data sources.

Besides the efficacy of the gene knockdown, a number of other factors confound our analysis, including the specificity and efficacy of cross-linking and immunoprecipitation (IP). The efficacy of RBP-RNA interaction assessment depends on the crosslinking method and varies for single-stranded and double-stranded RNAs (94). Besides this, the crosslinking position could itself be confounded by intramolecular base pairings, which often play a role in RNA processing (95), or some proteins within the size-matched control fraction of eCLIP could be not completely purified away (94). Also, it cannot be excluded that IP sample is contaminated by cross-linked interacting RPBs because they often control their targets combinatorially through interacting with closely located binding sites, or that multiple domains of the same factor independently make contacts with distinct portions of the pre-mRNA and the actual binding site could be different from the crosslinking position (96). Also, some of the observed discrepancies between eCLIP and shRNA-KD experiments could be due to the differences between human cell lines (K562 and HepG2 for eCLIP and RBP-KD, and HEK293 for NMD inactivation). As a result, eCLIP track may contain false positive peaks that represent the binding of the interacting partners or, conversely, some of the true eCLIP peaks could be missing.

According to Figure 1D, a negative feedback AS-NMD loop is characterized by the opposite reaction of its regulatory exons, poison or essential, to the inactivation of NMD pathway and to that of the RBP itself. Exons that react synergetically to both these perturbations suggest the existence of a similar positive feedback mechanism, which would work through repression of a poisonous exon, or activation of an essential exon in a manner opposite to that shown in Figures 1A and B. Unlike negative feedback loops, which tend to stabilize the output of gene regulatory circuits by compensatory changes in the direction opposite to the original deviation, positive feedback systems are less common, but have other features that are important in biological systems, including bistability, hysteresis and non-linear activation properties (97). An example of a positive feedback loop architecture at the transcriptional level is the regulatory network of four TFs of the bacterial DtxR family that maintains intracellular iron balance in archea (98). Considering that the global architecture of SF regulatory networks is quite different from that of transcription factors (in personal communication with Dr. S. Brenner), with more of a homeostatic role for SFs and more of a differentiating role for transcription factors, it remains an open question whether positive feedback loops at the post-transcriptional level exist at all.

However, we should note that regulatory mechanisms shown in Figure 1B and C operate at a special range of cellular concentrations, in which RBP is overexpressed, and there should be a non-linear, cooperative mechanism of activation or repression. For example, if an RBP is expressed at moderate levels and the cooperative mechanism in Figure 1B is not activated, then the inclusion rate of the poison exon should be low, and further decrease of the expression level will result in ΔΨ ≃ 0. This partially explains the lack of response in some RBPs with known AS-NMD mechanisms. Moreover, it is not uncommon for some RBPs to work as both splicing enhancers and silencers: at high concentrations it activates the inclusion of a poison exon, while at low concentrations it may become a suppressor. Therefore, the principle illustrated in Figure 1D must generally work at a high RBP concentration, and the presence of exons with synergetic ΔΨ changes is not contradictory.

The approach implemented here is based on the analysis of exon inclusion rates in two particular cases of poison and essential exons. We used the definition of exon inclusion rate Ψ that was originally introduced for cassette exons (59), but can also be interpreted in a broader sense for a larger class of splicing events, including alternative donor and acceptor site usage. A similar analysis of the Completeness of Splicing Index (59) can be used to identify regulatory intron retention cases, which are often associated with down-regulation of gene expression via NMD (99). In general, this methodology can be applied to arbitrary types of splicing events that are associated with downstream PTCs. However, such splicing events are often missing from the annotation databases because of undercoverage bias as a result of NMD degradation, and the computational identification of NMD transcripts is not completely straightforward. A similar approach can be applied for the discovery of cross-regulation of RBP expression, but this and many other follow-up questions go beyond the scope of this report.

## CONCLUSION

This paper presents an integrative bioinformatic analysis of a large panel of high-throughput binding and gene expression assays to find novel autoregulatory feedback loops of alternative splicing coupled with nonsense-mediated mRNA decay (AS-NMD). We confirm mechanistic scenarios that were proposed previously for SRSF7 and U2AF1 genes and identify two novel candidates, SFPQ and RPS3, which likely maintain their physiological concentrations via AS-NMD. Many other RBP genes may control their homeostasis by the same mechanism. We made the results of this study available through a UCSC Genome Browser track hub to be used for identification of post-transcriptional gene regulatory networks that operate through AS-NMD.

## Supplementary Material

gkz193_Supplemental_File
