# Supplementary material for: Integrative transcriptomic analysis suggests new autoregulatory splicing events coupled with nonsense-mediated mRNA decay
Source: Nucleic Acids Res. 2019 Feb 27;47(10):5293–306. doi: 10.1093/nar/gkz193 (PMC6547761; doi:10.1093/nar/gkz193)
Supplement: gkz193_Supplemental_File [file gkz193_supplemental_file.pdf]

Integrative transcriptomic analysis suggests new autoregulatory splicing events coupled with nonsense-mediated mRNA decay : SUPPLEMENTARY INFORMATION

D. Pervouchine *et al.*

February 1, 2019

List of Figures

S1	The distribution of exon z-scores	2
S2	Differential exon inclusion analysis in SMG6/XRN1 co-depletion	3
S3	Relative positions of reactive exons	4
S4	Frequencies of essential and non-essential internal exons	4
S5	Median distance to the closet cognate eCLIP peak	5
S6	Reactive exons with a nearby cognate eCLIP	6
	(A) FUS	6
	(B) HNRNPM	6
	(C) U2AF2	6
	(D) IGFBP2	6
S7	Examples in PTBP1 and TARDBP genes	7
	(A) PTBP1	7
	(B) TARDBP	7
S8	Reactive exons without nearby cognate eCLIP peaks	8
	(A) RBM39	8
	(B) SRSF3	8
	(C) SRSF4	8

List of Tables

S1	The list of accession numbers	12
S2	GO enrichment analysis of GWN	12
S3	The top 15 genes with the largest gene expression fold change	13
S4	GO enrichment analysis of NMD targets	13
S5	GO enrichment analysis of hosts of reactive exons	14

1 Supplementary Figures

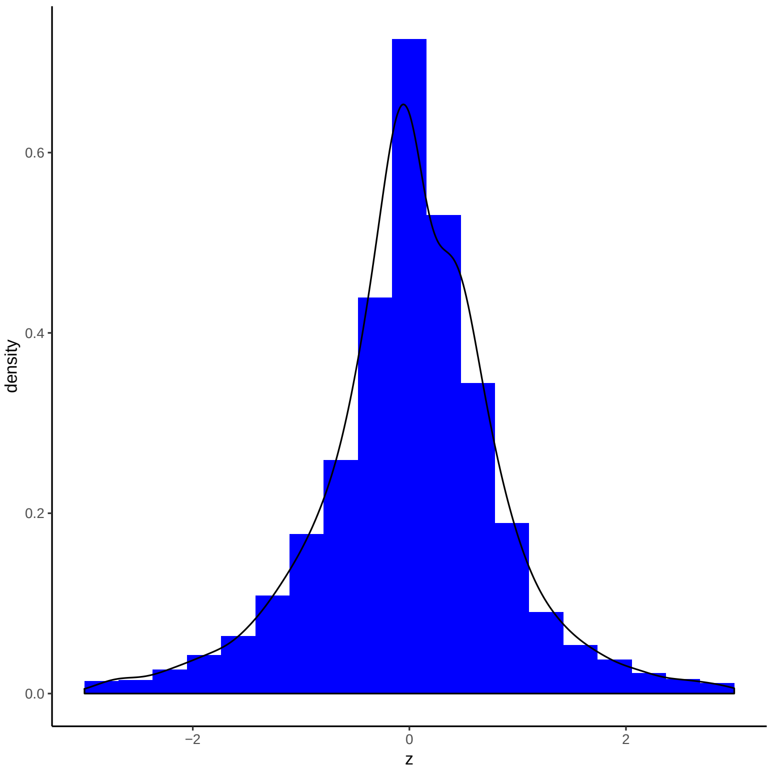

Figure S1: The distribution of exon z-scores. In each bin of $\log_{10}$ of the mean split read count, we computed the mean and the standard deviation of $\Delta\Psi$, excluding exons with $\Delta\Psi = 0$, and assigned the corresponding z-score to each exon.

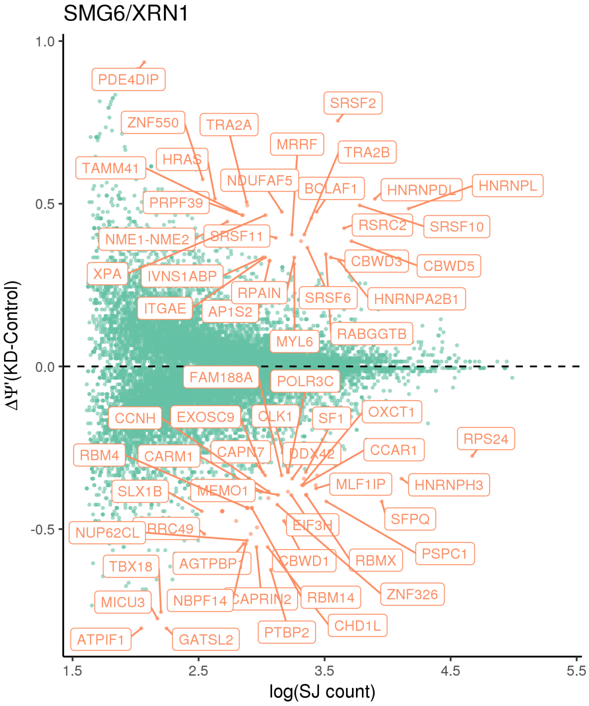

(A)

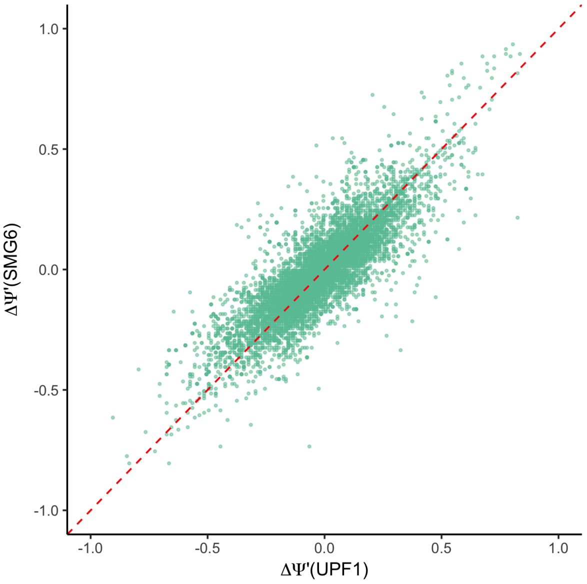

(B)

Figure S2: **(a)** Differential exon inclusion analysis in SMG6/XRN1 co-depletion. Changes at the 1% significance level are shown in orange. **(a)** The difference of exon inclusion ($\Delta\Psi$) in UPF1/XRN1 co-depletion vs. SMG6/XRN1 co-depletion.

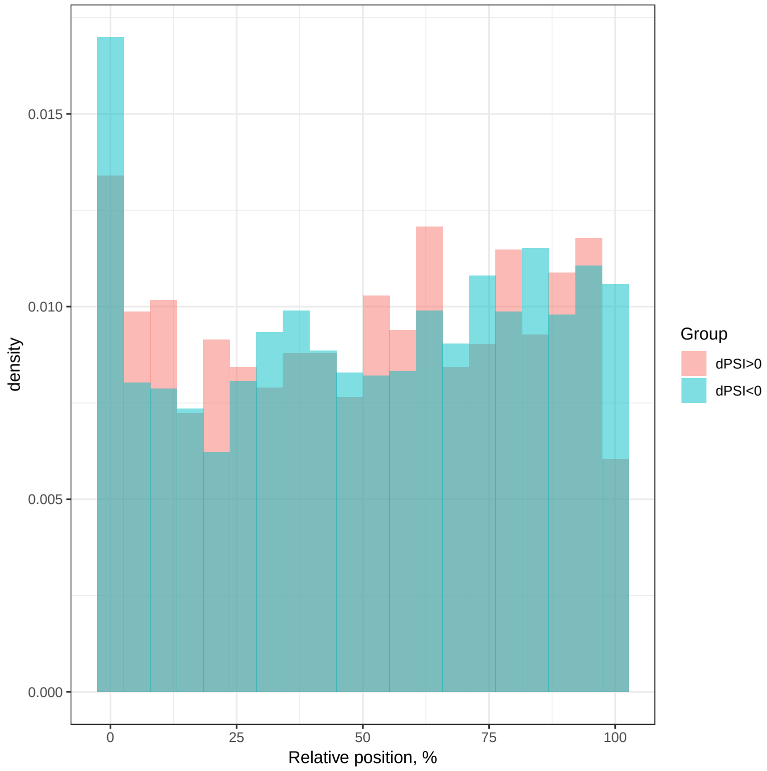

Figure S3: Relative positions within transcript of exons that are reactive to NMD inactivation.

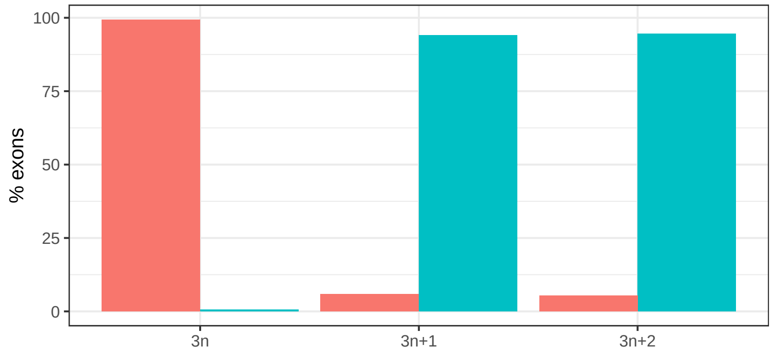

Figure S4: The percentage of essential (green) and non-essential (red) internal exons among all exons annotated in the GENCODE v19 database [1].

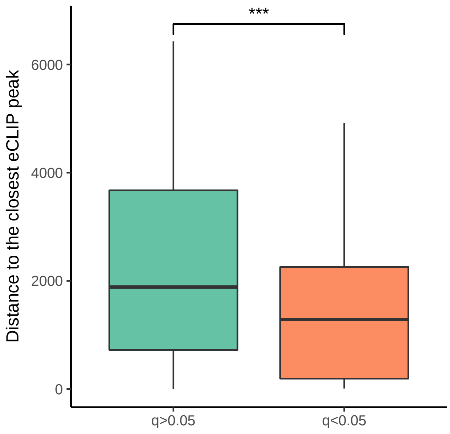

Figure S5: The median distance to the closet cognate eCLIP peak for exons that significantly change their inclusion level after NMD inactivation ($q < 0.05$) and that for the other exons ($q > 0.05$).

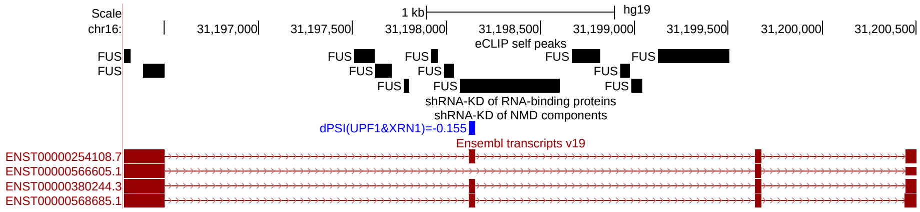

(A) FUS

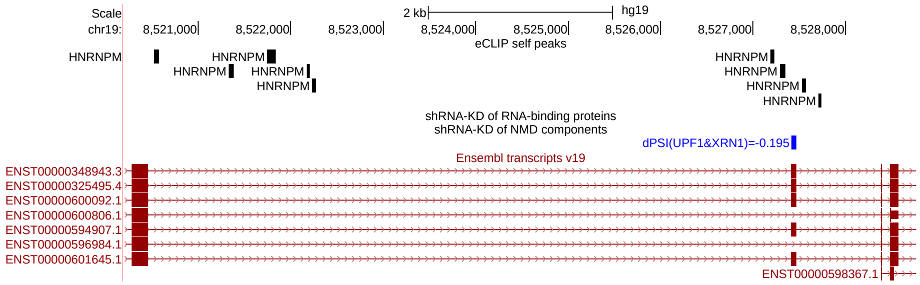

(B) HNRNPM

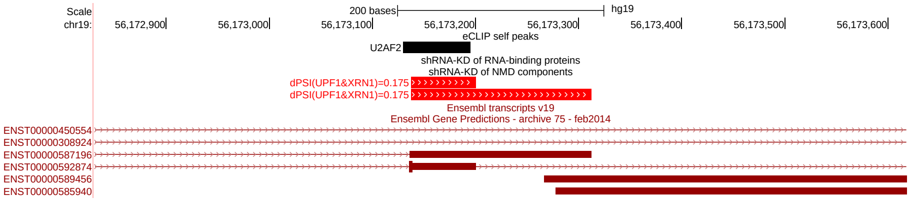

(C) U2AF2

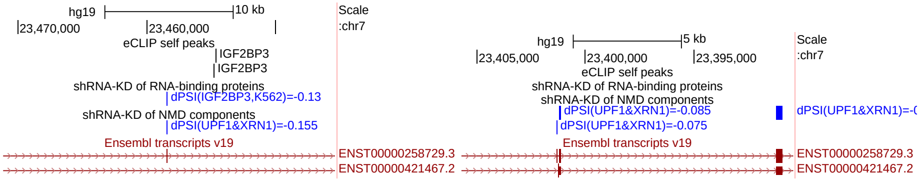

(D) IGFBP2

Figure S6: **(a)-(d)** Reactive exons with a nearby cognate eCLIP peak in FUS, HNRNPM, U2AF2, and IGFBP2 genes.

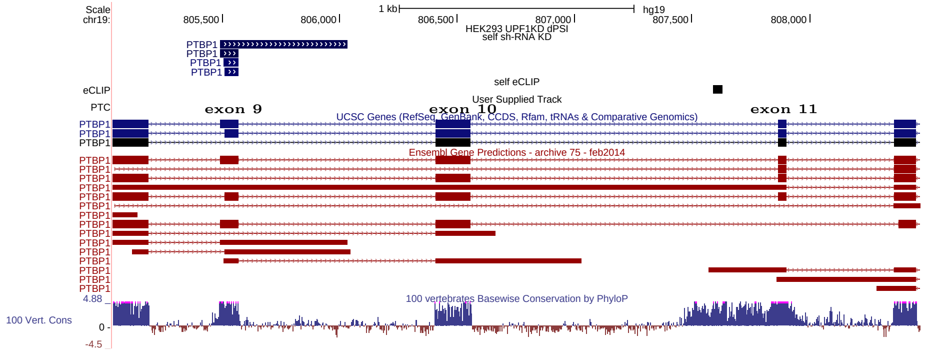

(A) PTBP1

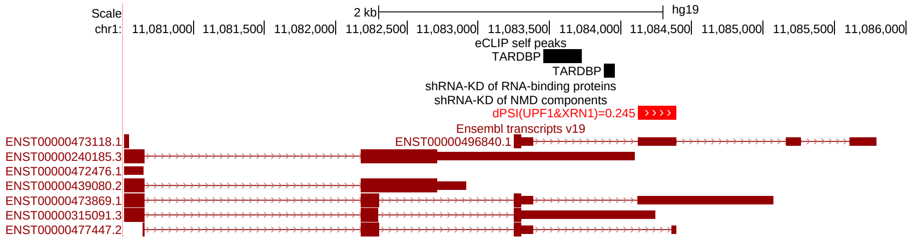

(B) TARDBP

Figure S7: **(a)** Essential exons in PTBP1 gene. The isoform with exon 11 skipping is not shown in Genome Browser. **(b)** TARDBP protein binds its 3'-UTR (binding sites are shown in black in the eCLIP track) and promotes splicing at poison exons.

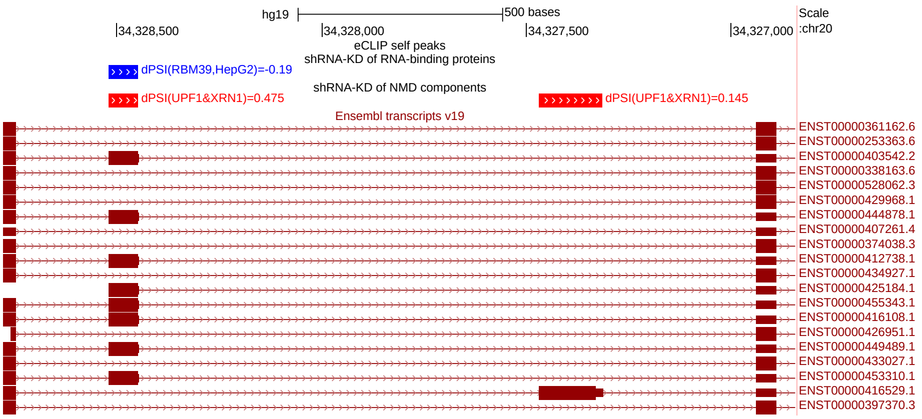

(A) RBM39

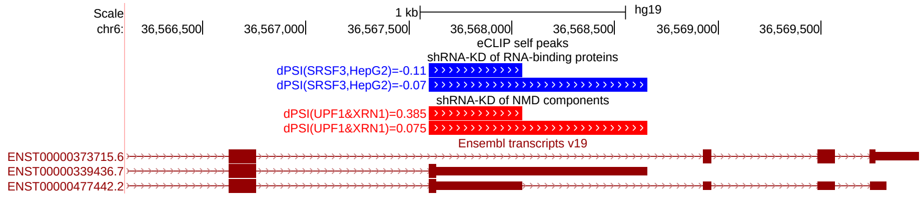

(B) SRSF3

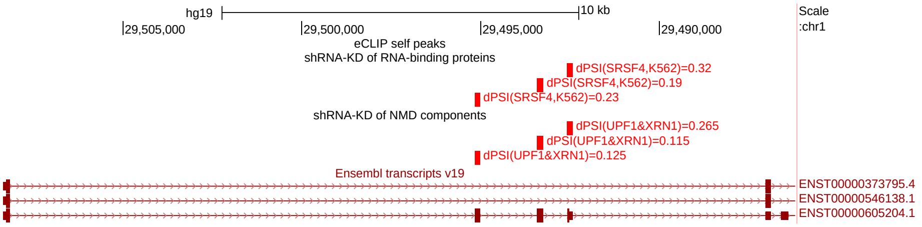

(C) SRSF4

Figure S8: **(a)-(c)** Reactive exons without nearby cognate eCLIP peaks in RBM39, SRSF3, and SRSF4 genes.

2 Supplementary Tables

cell	target	PEAKS1	PEAKS2	KD1	KD2	CONT1	CONT2
HepG2	AKAP1	807KPM	271UXH	446GNQ	554EIV	989TXQ	558ZFO
HepG2	BCCIP	698TRT	627YDB	085YWZ	813CJQ	572NPL	689YCR
HepG2	BCLAF1	691JPV	858HBB	878AAZ	587FCY	572NPL	689YCR
HepG2	BUD13	364QPG	929ITC	944UDR	526PAV	236XCF	901WHO
HepG2	CSTF2	892JUQ	370ZBJ	938DQM	866AHO	572NPL	689YCR
HepG2	CSTF2T	700HIR	786JAM	334UJK	529NFO	057JNE	294SKR
HepG2	DDX3X	798XQM	799ICU	572OTL	196CGJ	989TXQ	558ZFO
HepG2	DDX52	591WOA	539JUN	608NGH	653INZ	574DLW	847SRU
HepG2	DDX55	315AVG	584QHX	876ECR	193SMU	572NPL	689YCR
HepG2	DDX59	485MSN	441KPC	228NAK	802SDT	738YFT	293RIK
HepG2	DDX6	438KSN	650BQD	365JNK	725RWM	989TXQ	558ZFO
HepG2	DHX30	191HRQ	506NPG	751TAL	402UGA	784SML	487UNM
HepG2	DKC1	074ATJ	043XKO	572VRD	454QYL	566XUG	486GXG
HepG2	EFTUD2	511ZTQ	955RUR	039BLK	149WKD	574DLW	847SRU
HepG2	EIF3D	738CPK	650RVF	713UFE	708DON	574DLW	847SRU
HepG2	FAM120A	287ADK	511SXK	784QKI	143FVG	574DLW	847SRU
HepG2	FASTKD2	503DBN	136QLO	850TNI	330THZ	057JNE	294SKR
HepG2	FKBP4	993XGD	585PKG	982ALW	257YRE	572NPL	689YCR
HepG2	FTO	655JNU	925SGC	408FIB	098ONQ	738YFT	293RIK
HepG2	FUBP3	410ITK	714TFP	513ADH	880UOR	926DGG	718LIT
HepG2	G3BP1	934JFZ	165JYX	844ZHZ	529THE	989TXQ	558ZFO
HepG2	GRSF1	451GQU	733VOV	016WDI	506AFF	869IKC	996UTY
HepG2	GRWD1	735YNV	689GDA	420WYN	181DMT	261BLR	966QBV
HepG2	HNRNPA1	604WJZ	535MAE	350IPK	045OGG	784SML	487UNM
HepG2	HNRNPC	401SPQ	738FJS	181LSI	497PPO	784SML	487UNM
HepG2	HNRNPK	877KBJ	556EBT	899ZBQ	433BRP	989TXQ	558ZFO
HepG2	HNRNPL	581SSQ	415HDB	536UGZ	869LKX	566IXO	394YXV
HepG2	HNRNPM	040JSC	542FRX	601DUB	240HMJ	236XCF	901WHO
HepG2	HNRNPU	587FNW	024FCB	292RTX	516UIQ	784SML	487UNM
HepG2	HNRNPUL1	676GXG	801KHR	134YSM	596UTY	686VNL	430GWF
HepG2	IGF2BP3	175PMW	858ZLX	753NXB	441NPW	989TXQ	558ZFO
HepG2	ILF3	535COB	460MRE	485RWH	073SBH	869IKC	996UTY
HepG2	KHSRP	509GWT	891IAC	021RRG	310LTU	989TXQ	558ZFO
HepG2	LARP4	288SGB	130RAQ	890GSN	760RVL	930OYV	934UWJ
HepG2	LARP7	440FLF	699ZLU	870DWP	787GCZ	261BLR	966QBV
HepG2	LIN28B	976KYV	381VVY	511GIN	446IUZ	261BLR	966QBV
HepG2	LSM11	220ECM	870HZF	502SMN	939XZO	261BLR	966QBV
HepG2	MATR3	874BAL	333CUY	780SYO	246QJB	989TXQ	558ZFO
HepG2	NCBP2	047YUZ	500HBQ	594LYM	083LTV	989TXQ	558ZFO
HepG2	NIP7	077RVD	527MFS	354LYI	774DZT	261BLR	966QBV
HepG2	NKRF	864UGQ	233SNH	294MHK	100KDW	093UUE	870WUB
HepG2	NOL12	716SCR	515WRY	806ZKL	466PAF	566IXO	394YXV
HepG2	PCBP1	940CJE	252BJN	463AMS	174ZQQ	261RLR	834TYC
HepG2	PPIG	597JSM	603DSB	374SOY	585WPZ	093UUE	870WUB
HepG2	PRPF8	646XFI	156PFJ	727IHN	192LSC	572NPL	689YCR
HepG2	PTBP1	471GJE	154BAE	145TKU	855TDM	261RLR	834TYC
HepG2	QKI	459ANM	525PZA	354FHT	819VYL	261RLR	834TYC
HepG2	RBFOX2	639MYI	664WCU	880HKN	511KFD	686VNL	430GWF
HepG2	RBM15	017HYK	971BBY	276WQL	057WEZ	278EWB	376SXK
HepG2	RBM22	990UNN	353XWS	030OLZ	369NEZ	742NXL	340UAV

HepG2	SF3A3	642VTG	075FPZ	923LZN	677KRY	168UDP	321ZFN
HepG2	SF3B4	581RVY	363PGR	301YOD	784ZDW	261RLR	834TYC
HepG2	SFPQ	221WOF	919FIY	611BJW	402HCN	686VNL	430GWF
HepG2	SRSF1	179SCM	184TBM	856VSZ	942DHD	261RLR	834TYC
HepG2	SRSF7	774EDY	913UPA	642TXU	222WYC	261RLR	834TYC
HepG2	SRSF9	409GPI	125UVA	754SUW	664KDY	261RLR	834TYC
HepG2	SSB	746RLH	828WCR	647DVY	105XIV	742NXL	340UAV
HepG2	SUGP2	699DTX	187DUQ	434QDG	622PET	093UUE	870WUB
HepG2	SUPV3L1	882KLS	089NRQ	082CNV	734OAK	572NPL	689YCR
HepG2	TAF15	603JTU	063BTH	777VEO	531USK	261RLR	834TYC
HepG2	TBRG4	472CCD	454ZHL	328RQK	496DOM	093UUE	870WUB
HepG2	TIA1	055IIM	358SGV	298LYN	243YSW	261RLR	834TYC
HepG2	TIAL1	456DAU	473IIG	593CFB	273JPU	784SML	487UNM
HepG2	TROVE2	515SVK	029CYV	119ZCG	115CEE	574DLW	847SRU
HepG2	U2AF1	295GHA	078SCZ	163IXV	172LGC	236XCF	901WHO
HepG2	U2AF2	444TCK	523JWI	911EJE	821FZB	686VNL	430GWF
HepG2	UCHL5	305LRB	171KTG	432SEN	300CDS	572NPL	689YCR
HepG2	UPF1	483STL	848JHB	850ORL	819BTX	168UDP	321ZFN
HepG2	UTP18	227SZN	235ASG	977IXL	202TGW	168UDP	321ZFN
HepG2	XPO5	756YND	028MVM	386NUM	234HPE	869IKC	996UTY
HepG2	XRN2	944KMN	845EBP	450HWR	789QEA	572NPL	689YCR
HepG2	YBX3	904UKX	268SII	296FTT	036XRE	784SML	487UNM
K562	AARS	400HPZ	374VRV	893IIY	447ZJC	310XPL	301VBU
K562	AATF	715LDJ	415HBM	492MMG	127LYW	662KLY	356UJZ
K562	ABCF1	332BZR	376NBT	642YMU	494AFA	109UAW	827AVO
K562	AGGF1	450VBG	683KLO	548MMW	654OXL	221UUT	713YLH
K562	AKAP1	701BNI	972INE	527DFW	825OSD	451VCI	957JFM
K562	AKAP8L	189LGT	781FPH	321WXT	017QOH	221UUT	713YLH
K562	APOBEC3C	519AJS	319VBB	775RQD	544QYX	345DRD	171TGT
K562	AQR	325SFV	465QKS	392SMI	465UCM	971OQY	824KJT
K562	BUD13	102QYZ	560OAU	972TOM	559DCV	109UAW	827AVO
K562	CPEB4	379DBY	104VMP	960MRQ	360MMY	840VCF	306VXJ
K562	CPSF6	284QIO	515QAG	112VHC	885HMK	907AVE	006QUW
K562	CSTF2T	043QXI	379UQU	234KME	268PZN	662KLY	356UJZ
K562	DDX21	945XNS	572GMO	576IXB	964BVO	250NQT	072REC
K562	DDX24	006KFT	354NUG	661TWW	325OHE	451VCI	957JFM
K562	DDX3X	039BKT	737SOJ	303ZST	109RMW	451VCI	957JFM
K562	DDX51	475ZZT	042FAB	265DPE	197WZP	360OFU	269IZJ
K562	DDX52	343EUG	818KZJ	817BSA	935IWT	360OFU	269IZJ
K562	DDX55	432FVJ	099PFC	872MZO	109SXS	310XPL	301VBU
K562	DDX6	626UUD	906PBB	514XUK	997AWJ	451VCI	957JFM
K562	DHX30	507AKH	634UAJ	424VAD	867IZU	678FMA	965KEL
K562	DROSHA	602ZLI	840QVA	362VKF	382SWC	721YDQ	471LQQ
K562	EFTUD2	689YIJ	691BHS	758WPH	993VQJ	360OFU	269IZJ
K562	EIF3G	362BDI	258VPL	970NBJ	837MHH	951AKS	058RBW
K562	EIF4G2	462XFB	512MNG	960NRI	077BDV	345DRD	171TGT
K562	EWSR1	535SBV	621ZUN	154NAH	589TXX	451VCI	957JFM
K562	FAM120A	150KMB	901AAL	644NDS	132LJM	360OFU	269IZJ
K562	FASTKD2	885BWV	943JBN	781YOL	072TOC	951AKS	058RBW
K562	FMR1	965SEZ	393YLO	120YNR	365TMN	907AVE	006QUW
K562	FTO	465VTS	485IYX	139JVG	620KGA	907AVE	006QUW
K562	FUS	446LST	764SZR	829EPJ	436IYL	951AKS	058RBW
K562	FXR1	678MSG	464GTB	149PGZ	923OMI	451VCI	957JFM
K562	FXR2	871ZJM	639OOE	678VOG	293OGK	451VCI	957JFM

K562	GEMIN5	594WHT	083ZYT	633KQS	946XFO	360OFU	269IZJ
K562	GPKOW	760JZI	410KNT	454PAB	834RZF	907AVE	006QUW
K562	GRWD1	607PIM	757KMM	174EQN	074WVB	310XPL	301VBU
K562	GTF2F1	739CHZ	968DUU	388MKH	204XOP	662KLY	356UJZ
K562	HLTF	726GRF	952TOI	488HTU	594VBS	662KLY	356UJZ
K562	HNRNPA1	615KJU	174RLY	398HLD	979PHM	094PAZ	699LOO
K562	HNRNPC	062LWZ	971ZDB	614AMY	476PZV	310XPL	301VBU
K562	HNRNPK	079MYZ	769QNG	808LWD	519LDN	250NQT	072REC
K562	HNRNPL	796KSB	782DAY	638CZN	621OWD	840VCF	306VXJ
K562	HNRNPM	016LSY	462KTW	353DLI	380WYR	109UAW	827AVO
K562	HNRNPU	014BXV	960NDY	799PKF	616IYG	094PAZ	699LOO
K562	HNRNPUL1	734NGD	297PQZ	650THJ	032BIU	721YDQ	471LQQ
K562	IGF2BP1	435MEM	701YCW	275QEO	597IYH	250NQT	072REC
K562	IGF2BP2	968WTB	512QJT	377DNM	739FOM	250NQT	072REC
K562	ILF3	857MJR	821QFO	176YUC	605MVJ	678FMA	965KEL
K562	KHDRBS1	390ANM	052ABQ	703FOH	414ANM	678FMA	965KEL
K562	KHSRP	498QZQ	531NDZ	907ZKI	795LDC	250NQT	072REC
K562	LARP4	305LNT	544HGJ	586UQY	393WYS	721YDQ	471LQQ
K562	LARP7	017NRG	208AAW	330TDS	616HGG	310XPL	301VBU
K562	LIN28B	823WIW	823UGV	385ZPU	505JCB	310XPL	301VBU
K562	LSM11	425PPQ	136HXF	200DJS	217NHJ	310XPL	301VBU
K562	MATR3	121ZEY	904GOE	947WJB	547XWI	250NQT	072REC
K562	METAP2	727RJA	041HMB	426HXK	532SHV	907AVE	006QUW
K562	MTPAP	984DXU	448TUI	504BUN	287RFQ	055IEE	823LBA
K562	NCBP2	509XPD	014NHI	342FMY	982FDC	451VCI	957JFM
K562	NONO	174MVU	201YQC	802XVR	687USH	360OFU	269IZJ
K562	NPM1	743GIA	392QAQ	838WJE	986JGI	721YDQ	471LQQ
K562	NSUN2	270MTA	257SMG	206WPX	678LZA	055IEE	823LBA
K562	PABPC4	463ZSJ	772SPP	979EOV	206ASM	812XUV	201SIR
K562	PCBP1	022GSN	381ZBA	644HYW	227XAW	250NQT	072REC
K562	PHF6	016IHL	967JAT	138JQO	340ZEG	310XPL	301VBU
K562	PPIL4	036ZXE	188XKC	085VCF	805KGC	689TGX	486RUF
K562	PRPF8	576DYQ	687LRO	096IJH	660WGY	360OFU	269IZJ
K562	PTBP1	262NXW	200AWX	813ICC	936ZPB	250NQT	072REC
K562	PUM1	933SBL	060XYT	463YXB	553SKC	345DRD	171TGT
K562	PUM2	091HXE	262JRE	735KTG	654RKB	345DRD	171TGT
K562	PUS1	990JXJ	268XIE	775UPA	722UNY	221UUT	713YLH
K562	QKI	534JXG	963AMI	124CQK	226JJS	812XUV	201SIR
K562	RBFOX2	076RDA	994PFX	146CHN	920LNP	678FMA	965KEL
K562	RBM15	715SCF	404OSK	430ISK	355EHE	221UUT	713YLH
K562	RBM22	074GNZ	020UVE	856EUQ	210USC	250NQT	072REC
K562	RPS3	323ZDL	922WMS	580UQE	664HRD	951AKS	058RBW
K562	SAFB2	273BVZ	801ARQ	605NAW	198QMX	192WNC	546CWI
K562	SBDS	873TSY	175EIG	964ACB	055WUU	689TGX	486RUF
K562	SERBP1	979XAE	710IZS	579KTN	145RMT	360OFU	269IZJ
K562	SF3B1	498GFT	210VRR	888GPL	299OPQ	907AVE	006QUW
K562	SF3B4	013ZHF	658CBT	454UXB	730MBP	812XUV	201SIR
K562	SLBP	397VAA	874WRG	741VFO	579RKL	678FMA	965KEL
K562	SLTM	052VSB	392CBU	738RJJ	551BGC	951AKS	058RBW
K562	SMNDC1	581FUT	142PZD	755KEU	152IUL	662KLY	356UJZ
K562	SND1	479VZE	677MQI	521QBU	868JUI	951AKS	058RBW
K562	SRSF1	609IXO	287KWV	451ILH	446IVL	250NQT	072REC
K562	SRSF7	090BZK	939AMV	495CHO	871XKQ	250NQT	072REC
K562	SSB	704NUD	871PJK	150PMH	629VLO	951AKS	058RBW

K562	SUPV3L1	207VRH	942LSK	710LUV	654PVK	360OFU	269IZJ
K562	TAF15	979IVI	783IGF	669KZU	862NET	812XUV	201SIR
K562	TARDBP	697CMV	333KPU	782EXB	202XMY	250NQT	072REC
K562	TBRG4	033ZLA	040CEA	374VZI	341NGU	812XUV	201SIR
K562	TIA1	509PKA	673MNH	622BGG	675IKK	250NQT	072REC
K562	TRA2A	257VRQ	744EQP	120IKS	995OUG	250NQT	072REC
K562	TROVE2	243GNO	043TCO	857CHR	659AIM	892EVK	680FLW
K562	U2AF1	555DWV	468LUS	352HEO	049WEE	812XUV	201SIR
K562	U2AF2	430RTZ	454FEB	942VXX	676CZA	812XUV	201SIR
K562	UCHL5	713NFZ	912GTJ	922PAN	658NSQ	360OFU	269IZJ
K562	UPF1	873FZT	792HYY	243KYH	632FEO	055IEE	823LBA
K562	UTP18	379LHC	014AWF	191GKG	609RPE	221UUT	713YLH
K562	WDR3	991NPH	380SYQ	668TYJ	450FFU	055IEE	823LBA
K562	WDR43	425HAU	509FAF	799ITP	605UTR	055IEE	823LBA
K562	WRN	138IUK	109LYL	406QFV	354ESP	971OQY	824KJT
K562	XRCC6	564IUM	007DHU	175FUM	180LMY	662KLY	356UJZ
K562	XRN2	441GJR	167DBG	950IVB	092ZPJ	360OFU	269IZJ
K562	YBX3	526YVR	231IID	048SVW	507VIL	678FMA	965KEL
K562	ZC3H8	515ATI	990BSZ	098NLK	635XIV	971OQY	824KJT
K562	ZRANB2	464HWD	709ENB	586GDJ	031QIE	662KLY	356UJZ

Table S1: The list of RNA-binding proteins, eCLIP (PEAK1 and PEAK2), shRNA-KD followed RNA-seq experiment (KD1, KD2) and their control experiments (CONTROL1, CONTROL2) accession numbers [2, 3]. The "ENCFF" prefix in accession has been omited.

Table S2: GO enrichment analysis (Biological Process) of Genes with at least one NMD transcript (GWN) using GOrilla [4].

GO Term	Description	p-value	FDR q-value
GO:0003824	catalytic activity	1.04E-39	4.27E-36
GO:0036094	small molecule binding	3.91E-23	8.01E-20
GO:1901265	nucleoside phosphate binding	9.60E-23	1.31E-19
GO:0000166	nucleotide binding	1.29E-22	1.32E-19
GO:0005524	ATP binding	3.56E-16	2.92E-13
GO:0005488	binding	1.22E-15	8.34E-13
GO:0035639	purine ribonucleoside triphosphate binding	1.50E-15	8.81E-13
GO:0003723	RNA binding	1.59E-15	8.15E-13
GO:0030554	adenyl nucleotide binding	1.90E-15	8.67E-13
GO:0032559	adenyl ribonucleotide binding	2.00E-15	8.21E-13
GO:0032549	ribonucleoside binding	3.31E-15	1.24E-12
GO:0001883	purine nucleoside binding	3.69E-15	1.26E-12
GO:0032550	purine ribonucleoside binding	4.11E-15	1.30E-12
GO:0001882	nucleoside binding	5.01E-15	1.47E-12
GO:0005515	protein binding	6.46E-15	1.77E-12

Table S3: The top 15 genes with the largest gene expression fold change between UPF1/XRN1 co-depletion and control.

ID	baseMean	log2FoldChange	p-value	Gene
TCONS_00166954	5365.64	2.562	0.01388	BRD2
TCONS_00034807	18856.60	2.539	0.01384	SNHG1
TCONS_00176132	8304.77	2.534	0.01429	CCT6P3
TCONS_00176130	3356.49	2.525	0.01538	CCT6P3
TCONS_00001342	7025.46	2.505	0.01522	SRRM1
TCONS_00070942	3337.18	2.505	0.01596	61E3.4
TCONS_00159029	3264.56	2.498	0.01620	MATR3
TCONS_00070925	2505.97	2.493	0.01679	SMG1P1
TCONS_00019843	2258.43	2.492	0.01721	AGAP6
TCONS_00163703	2612.10	2.484	0.01699	FAM13B
TCONS_00101194	4421.11	2.478	0.01646	SAE1
TCONS_00182764	2072.64	2.478	0.01765	DNAJC2
TCONS_00127428	9299.07	2.478	0.01589	RBM39
TCONS_00010353	2930.28	2.477	0.01796	CROCCP2
TCONS_00198400	2045.45	2.477	0.01773	FBXW2

Table S4: GO enrichment analysis (Biological Process) of the 200 genes with the largest fold change between UPF1/XRN1 co-depletion and control. Top 10 GO terms with P-value $< 10^{-3}$ are shown.

GOBPID	ExpCount	Count	Size	Term
GO:0006396	7	37	936	RNA processing
GO:0006397	3	27	491	mRNA processing
GO:0008380	3	23	428	RNA splicing
GO:0016071	6	30	812	mRNA metabolic process
GO:0000377	2	19	323	RNA splicing, via transesterification reactions with bulged adenosine as nucleophile
GO:0000398	2	19	323	mRNA splicing, via spliceosome
GO:0000375	2	19	326	RNA splicing, via transesterification reactions
GO:0090304	37	65	5206	nucleic acid metabolic process
GO:0050684	1	9	112	regulation of mRNA processing
GO:0043484	0	6	118	regulation of RNA splicing

Table S5: GO enrichment analysis (Biological Process) of the genes hosting differentially spliced exons (adjusted p-value< 0.05) in UPF1/XRN1 co-depletion and control. The top 15 GO terms with P-value$< 10^{-3}$ are shown.

GOBPID	OddsRatio	ExpCount	Count	Size	Term
GO:0043484	30.773	0	6	118	regulation of RNA splicing
GO:0033119	103.119	0	4	25	negative regulation of RNA splicing
GO:0006396	8.886	2	12	936	RNA processing
GO:0000377	15.447	1	8	323	RNA splicing, via transesterification reactions with bulged adenosine as nucleophile
GO:0000398	15.447	1	8	323	mRNA splicing, via spliceosome
GO:0000375	15.298	1	8	326	RNA splicing, via transesterification reactions
GO:0048024	38.162	0	5	78	regulation of mRNA splicing, via spliceosome
GO:0008380	11.514	1	8	428	RNA splicing
GO:0050684	25.984	0	5	112	regulation of mRNA processing
GO:0016071	7.934	2	10	812	mRNA metabolic process
GO:0006397	9.975	1	8	491	mRNA processing
GO:0048025	92.663	0	3	20	negative regulation of mRNA splicing, via spliceosome
GO:0050686	58.310	0	3	30	negative regulation of mRNA processing
GO:0006406	20.329	0	4	110	mRNA export from nucleus
GO:0071427	20.329	0	4	110	mRNA-containing ribonucleoprotein complex export from nucleus

SupplementaryDataFie 1: SupplementaryDataFile1.tsv

SupplementaryDataFie 2: SupplementaryDataFile2.tsv

SupplementaryDataFie 3: SupplementaryDataFile3.tsv

Supplementary References

[1] Church, D. M., Schneider, V. A., Graves, T., Auger, K., Cunningham, F., Bouk, N., Chen, H. C., Agarwala, R., McLaren, W. M., Ritchie, G. R., Albracht, D., Kremitzki, M., Rock, S., Kotkiewicz, H., Kremitzki, C., Wollam, A., Trani, L., Fulton, L., Fulton, R., Matthews, L., Whitehead, S., Chow, W., Torrance, J., Dunn, M., Harden, G., Threadgold, G., Wood, J., Collins, J., Heath, P., Griffiths, G., Pelan, S., Grafham, D., Eichler, E. E., Weinstock, G., Mardis, E. R., Wilson, R. K., Howe, K., Flicek, P., and Hubbard, T. (Jul, 2011) Modernizing reference genome assemblies. *PLoS Biol.,* **9**(7), e1001091.

[2] Dunham, I., Kundaje, A., Aldred, S. F., Collins, P. J., Davis, C. A., Doyle, F., Epstein, C. B., Frietze, S., Harrow, J., Kaul, R., Khatun, J., Lajoie, B. R., Landt, S. G., Lee, B. K., Pauli, F., Rosenbloom, K. R., Sabo, P., Safi, A., Sanyal, A., Shoresh, N., Simon, J. M., Song, L., Trinklein, N. D., Altshuler, R. C., Birney, E., Brown, J. B., Cheng, C., Djebali, S., Dong, X., Dunham, I., Ernst, J., Furey, T. S., Gerstein, M., Giardine, B., Greven, M., Hardison, R. C., Harris, R. S., Herrero, J., Hoffman, M. M., Iyer, S., Kellis, M., Khatun, J., Kheradpour, P., Kundaje, A., Lassmann, T., Li, Q., Lin, X., Marinov, G. K., Merkel, A., Mortazavi, A., Parker, S. C., Reddy, T. E., Rozowsky, J., Schlesinger, F., Thurman, R. E., Wang, J., Ward, L. D., Whitfield, T. W., Wilder, S. P., Wu, W., Xi, H. S., Yip, K. Y., Zhuang, J., Pazin, M. J., Lowdon, R. F., Dillon, L. A., Adams, L. B., Kelly, C. J., Zhang, J., Wexler, J. R., Green, E. D., Good, P. J., Feingold, E. A., Bernstein, B. E., Birney, E., Crawford, G. E., Dekker, J., Elnitski, L., Farnham, P. J., Gerstein, M., Giddings, M. C., Gingeras, T. R., Green, E. D., Guigo, R., Hardison, R. C., Hubbard, T. J., Kellis, M., Kent, W., Lieb, J. D., Margulies, E. H., Myers, R. M., Snyder, M., Stamatoyannopoulos, J. A., Tenenbaum, S. A., Weng, Z., White, K. P., Wold, B., Khatun, J., Yu, Y., Wrobel, J., Risk, B. A., Gunawardena, H. P., Kuiper, H. C., Maier, C. W., Xie, L., Chen, X., Giddings, M. C., Bernstein, B. E., Epstein, C. B., Shoresh, N., Ernst, J., Kheradpour, P., Mikkelsen, T. S., Gillespie, S., Goren, A., Ram, O., Zhang, X., Wang, L., Issner, R., Coyne, M. J., Durham, T., Ku, M., Truong, T., Ward, L. D., Altshuler, R. C., Eaton, M. L., Kellis, M., Djebali, S., Davis, C. A., Merkel, A., Dobin, A., Lassmann, T., Mortazavi, A., Tanzer, A., Lagarde, J., Lin, W., Schlesinger, F., Xue, C., Marinov, G. K., Khatun, J., Williams, B. A., Zaleski, C., Rozowsky, J., Roder, M., Kokocinski, F., Abdelhamid, R. F., Alioto, T., Antoshechkin, I., Baer, M. T., Batut, P., Bell, I., Bell, K., Chakrabortty, S., Chen, X., Chrast, J., Curado, J., Derrien, T., Drenkow, J., Dumais, E., Dumais, J., Duttagupta, R., Fastuca, M., Fejes-Toth, K., Ferreira, P., Foissac, S., Fullwood, M. J., Gao, H., Gonzalez, D., Gordon, A., Gunawardena, H. P., Howald, C., Jha, S., Johnson, R., Kapranov, P., King, B., Kingswood, C., Li, G., Luo, O. J., Park, E., Preall, J. B., Presaud, K., Ribeca, P., Risk, B. A., Robyr, D., Ruan, X., Sammeth, M., Sandhu, K. S., Schaeffer, L., See, L. H., Shahab, A., Skancke, J., Suzuki, A. M., Takahashi, H., Tilgner, H., Trout, D., Walters, N., Wang, H., Wrobel, J., Yu, Y., Hayashizaki, Y., Harrow, J., Gerstein, M., Hubbard, T. J., Reymond, A., Antonarakis, S. E., Hannon, G. J., Giddings, M. C., Ruan, Y., Wold, B., Carninci, P., Guigo, R., Gingeras, T. R., Rosenbloom,

K. R., Sloan, C. A., Learned, K., Malladi, V. S., Wong, M. C., Barber, G. P., Cline, M. S., Dreszer, T. R., Heitner, S. G., Karolchik, D., Kent, W., Kirkup, V. M., Meyer, L. R., Long, J. C., Maddren, M., Raney, B. J., Furey, T. S., Song, L., Grasfeder, L. L., Giresi, P. G., Lee, B. K., Battenhouse, A., Sheffield, N. C., Simon, J. M., Showers, K. A., Safi, A., London, D., Bhinge, A. A., Shestak, C., Schaner, M. R., Kim, S. K., Zhang, Z. Z., Mieczkowski, P. A., Mieczkowska, J. O., Liu, Z., McDaniell, R. M., Ni, Y., Rashid, N. U., Kim, M. J., Adar, S., Zhang, Z., Wang, T., Winter, D., Keefe, D., Birney, E., Iyer, V. R., Lieb, J. D., Crawford, G. E., Li, G., Sandhu, K. S., Zheng, M., Wang, P., Luo, O. J., Shahab, A., Fullwood, M. J., Ruan, X., Ruan, Y., Myers, R. M., Pauli, F., Williams, B. A., Gertz, J., Marinov, G. K., Reddy, T. E., Vielmetter, J., Partridge, E., Trout, D., Varley, K. E., Gasper, C., Bansal, A., Pepke, S., Jain, P., Amrhein, H., Bowling, K. M., Anaya, M., Cross, M. K., King, B., Muratet, M. A., Antoshechkin, I., Newberry, K. M., McCue, K., Nesmith, A. S., Fisher-Aylor, K. I., Pusey, B., DeSalvo, G., Parker, S. L., Balasubramanian, S., Davis, N. S., Meadows, S. K., Eggleston, T., Gunter, C., Newberry, J., Levy, S. E., Absher, D. M., Mortazavi, A., Wong, W. H., Wold, B., Blow, M. J., Visel, A., Pennachio, L. A., Elnitski, L., Margulies, E. H., Parker, S. C., Petrykowska, H. M., Abyzov, A., Aken, B., Barrell, D., Barson, G., Berry, A., Bignell, A., Boychenko, V., Bussotti, G., Chrast, J., Davidson, C., Derrien, T., Despacio-Reyes, G., Diekhans, M., Ezkurdia, I., Frankish, A., Gilbert, J., Gonzalez, J. M., Griffiths, E., Harte, R., Hendrix, D. A., Howald, C., Hunt, T., Jungreis, I., Kay, M., Khurana, E., Kokocinski, F., Leng, J., Lin, M. F., Loveland, J., Lu, Z., Manthravadi, D., Mariotti, M., Mudge, J., Mukherjee, G., Notredame, C., Pei, B., Rodriguez, J. M., Saunders, G., Sboner, A., Searle, S., Sisu, C., Snow, C., Steward, C., Tanzer, A., Tapanari, E., Tress, M. L., van Baren, M. J., Walters, N., Washietl, S., Wilming, L., Zadissa, A., Zhang, Z., Brent, M., Haussler, D., Kellis, M., Valencia, A., Gerstein, M., Reymond, A., Guigo, R., Harrow, J., Hubbard, T. J., Landt, S. G., Frietze, S., Abyzov, A., Addleman, N., Alexander, R. P., Auerbach, R. K., Balasubramanian, S., Bettinger, K., Bhardwaj, N., Boyle, A. P., Cao, A. R., Cayting, P., Charos, A., Cheng, Y., Cheng, C., Eastman, C., Euskirchen, G., Fleming, J. D., Grubert, F., Habegger, L., Hariharan, M., Harmanci, A., Iyengar, S., Jin, V. X., Karczewski, K. J., Kasowski, M., Lacroute, P., Lam, H., Lamarre-Vincent, N., Leng, J., Lian, J., Lindahl-Allen, M., Min, R., Miotto, B., Monahan, H., Moqtaderi, Z., Mu, X. J., O'Geen, H., Ouyang, Z., Patacsil, D., Pei, B., Raha, D., Ramirez, L., Reed, B., Rozowsky, J., Sboner, A., Shi, M., Sisu, C., Slifer, T., Witt, H., Wu, L., Xu, X., Yan, K. K., Yang, X., Yip, K. Y., Zhang, Z., Struhl, K., Weissman, S. M., Gerstein, M., Farnham, P. J., Snyder, M., Tenenbaum, S. A., Penalva, L. O., Doyle, F., Karmakar, S., Landt, S. G., Bhanvadia, R. R., Choudhury, A., Domanus, M., Ma, L., Moran, J., Patacsil, D., Slifer, T., Victorsen, A., Yang, X., Snyder, M., Auer, T., Centanin, L., Eichenlaub, M., Gruhl, F., Heermann, S., Hoeckendorf, B., Inoue, D., Kellner, T., Kirchmaier, S., Mueller, C., Reinhardt, R., Schertel, L., Schneider, S., Sinn, R., Wittbrodt, B., Wittbrodt, J., Weng, Z., Whitfield, T. W., Wang, J., Collins, P. J., Aldred, S. F., Trinklein, N. D., Partridge, E. C., Myers, R. M., Dekker, J., Jain, G., Lajoie, B. R., Sanyal, A., Balasundaram, G., Bates, D. L., Byron, R., Canfield, T. K., Diegel, M. J., Dunn, D., Ebersol, A. K., Frum, T., Garg, K., Gist, E., Hansen, R., Boatman, L., Haugen, E., Humbert, R., Jain,

G., Johnson, A. K., Johnson, E. M., Kutyavin, T. V., Lajoie, B. R., Lee, K., Lotakis, D., Maurano, M. T., Neph, S. J., Neri, F. V., Nguyen, E. D., Qu, H., Reynolds, A. P., Roach, V., Rynes, E., Sabo, P., Sanchez, M. E., Sandstrom, R. S., Sanyal, A., Shafer, A. O., Stergachis, A. B., Thomas, S., Thurman, R. E., Vernot, B., Vierstra, J., Vong, S., Wang, H., Weaver, M. A., Yan, Y., Zhang, M., Akey, J. M., Bender, M., Dorschner, M. O., Groudine, M., MacCoss, M. J., Navas, P., Stamatoyannopoulos, G., Kaul, R., Dekker, J., Stamatoyannopoulos, J. A., Dunham, I., Beal, K., Brazma, A., Flicek, P., Herrero, J., Johnson, N., Keefe, D., Lukk, M., Luscombe, N. M., Sobral, D., Vaquerizas, J. M., Wilder, S. P., Batzoglou, S., Sidow, A., Hussami, N., Kyriazopoulou-Panagiotopoulou, S., Libbrecht, M. W., Schaub, M. A., Kundaje, A., Hardison, R. C., Miller, W., Giardine, B., Harris, R. S., Wu, W., Bickel, P. J., Banfai, B., Boley, N. P., Brown, J. B., Huang, H., Li, Q., Li, J. J., Noble, W. S., Bilmes, J. A., Buske, O. J., Hoffman, M. M., Sahu, A. D., Kharchenko, P. V., Park, P. J., Baker, D., Taylor, J., Weng, Z., Iyer, S., Dong, X., Greven, M., Lin, X., Wang, J., Xi, H. S., Zhuang, J., Gerstein, M., Alexander, R. P., Balasubramanian, S., Cheng, C., Harmanci, A., Lochovsky, L., Min, R., Mu, X. J., Rozowsky, J., Yan, K. K., Yip, K. Y., and Birney, E. (Sep, 2012) An integrated encyclopedia of DNA elements in the human genome. *Nature,* **489**(7414), 57–74.

[3] Sloan, C. A., Chan, E. T., Davidson, J. M., Malladi, V. S., Strattan, J. S., Hitz, B. C., Gabdank, I., Narayanan, A. K., Ho, M., Lee, B. T., Rowe, L. D., Dreszer, T. R., Roe, G., Podduturi, N. R., Tanaka, F., Hong, E. L., and Cherry, J. M. (Jan, 2016) ENCODE data at the ENCODE portal. *Nucleic Acids Res.,* **44**(D1), D726–732.

[4] Eden, E., Navon, R., Steinfeld, I., Lipson, D., and Yakhini, Z. (Feb, 2009) GOrilla: a tool for discovery and visualization of enriched GO terms in ranked gene lists. *BMC Bioinformatics,* **10**, 48.
